# Supplementary material for: The Temporal Dynamics of Arc Expression Regulate Cognitive Flexibility
Source: Neuron. 2018 Jun 27;98(6):1124–1132.e7. doi: 10.1016/j.neuron.2018.05.012 (PMC6030446; doi:10.1016/j.neuron.2018.05.012)
Supplement: Document S1. Figures S1–S4 [file mmc1.pdf]

Neuron, Volume *98*

Supplemental Information

The Temporal Dynamics of Arc Expression Regulate Cognitive Flexibility

Mark J. Wall, Dawn R. Collins, Samantha L. Chery, Zachary D. Allen, Elissa D. Pastuzyn, Arlene J. George, Viktoriya D. Nikolova, Sheryl S. Moy, Benjamin D. Philpot, Jason D. Shepherd, Jürgen Müller, Michael D. Ehlers, Angela M. Mabb, and Sonia A.L. Corrêa

Figure S1

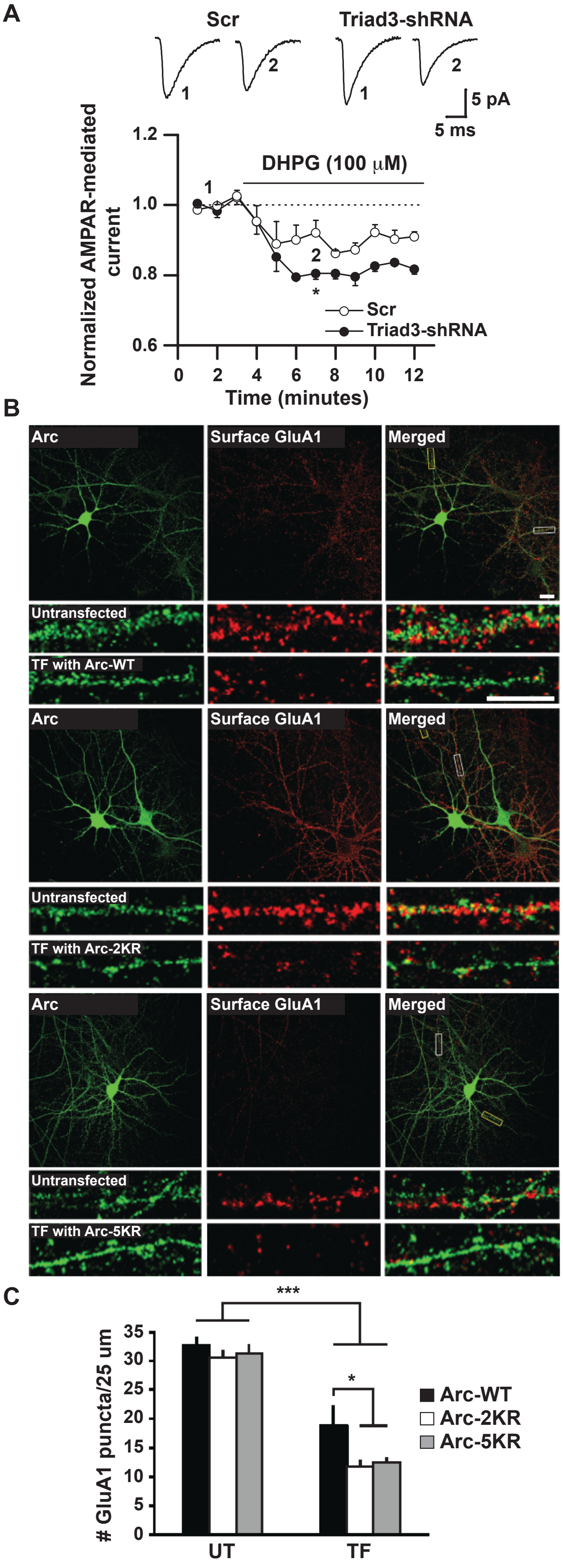
A
Scr
Triad3-shRNA
1
2
1
2
5 pA
5 ms
Normalized AMPAR-mediated current
1.2
1.0
0.8
0.6
DHPG (100 μM)
1
2
*
Scr
Triad3-shRNA
0
2
4
6
8
10
12
Time (minutes)
B
Arc
Surface GluA1
Merged
Untransfected
TF with Arc-WT
Arc
Surface GluA1
Merged
Untransfected
TF with Arc-2KR
Arc
Surface GluA1
Merged
Untransfected
TF with Arc-5KR
C
***
*
GluA1 puncta/25 um
35
30
25
20
15
10
5
0
UT
TF
Arc-WT
Arc-2KR
Arc-5KR

Figure S1. ArcKR expression enhances GluA1 internalization, Related to Figure 1

(A) Graph plotting normalized mean AMPA receptor-dependent mEPSC amplitudes against time for hippocampal cultured neurons expressing either *Triad3/Rnf216*-shRNA (n = 5) or scrambled RNA (n = 4) from at least 2 different culture preparations. Treatment with DHPG (100 μM) significantly enhances DHPG-LTD in *Triad3/Rnf216*-shRNA compared to neurons expressing scrambled RNA (measured at 6-7 min in DHPG compared to baseline, p = 0.026). *Top*, representative averaged mEPSC waveforms recorded at baseline and during DHPG exposure at times indicated in the plot (1, 1-2 min and 2, 6-7 min). Averages were constructed from at least 50 mEPSCs which were aligned on 50% of the rising phase. (B) Cultured cortical neurons (DIV 15) were transfected with Arc-WT (upper panel), Arc-2KR (middle), or Arc-5KR (bottom) constructs. Neurons were stained for surface GluA1 and Arc 16 h after transfection. As expected, overexpression of Arc-WT reduced surface GluA1 puncta compared to untransfected neurons. Zoomed panels, dendrites from untransfected (white box) and transfected (yellow box) neurons. Scale bars = 10 μm. (C) Overexpression of the Arc-KR constructs resulted in significant reduction in surface GluA1 puncta compared to Arc-WT overexpression. *n* = 2 dendrites/neuron, 15 dendrites/group. *p < 0.05; ***p < 0.001. Values represent mean ± S.E.M.

Figure S2

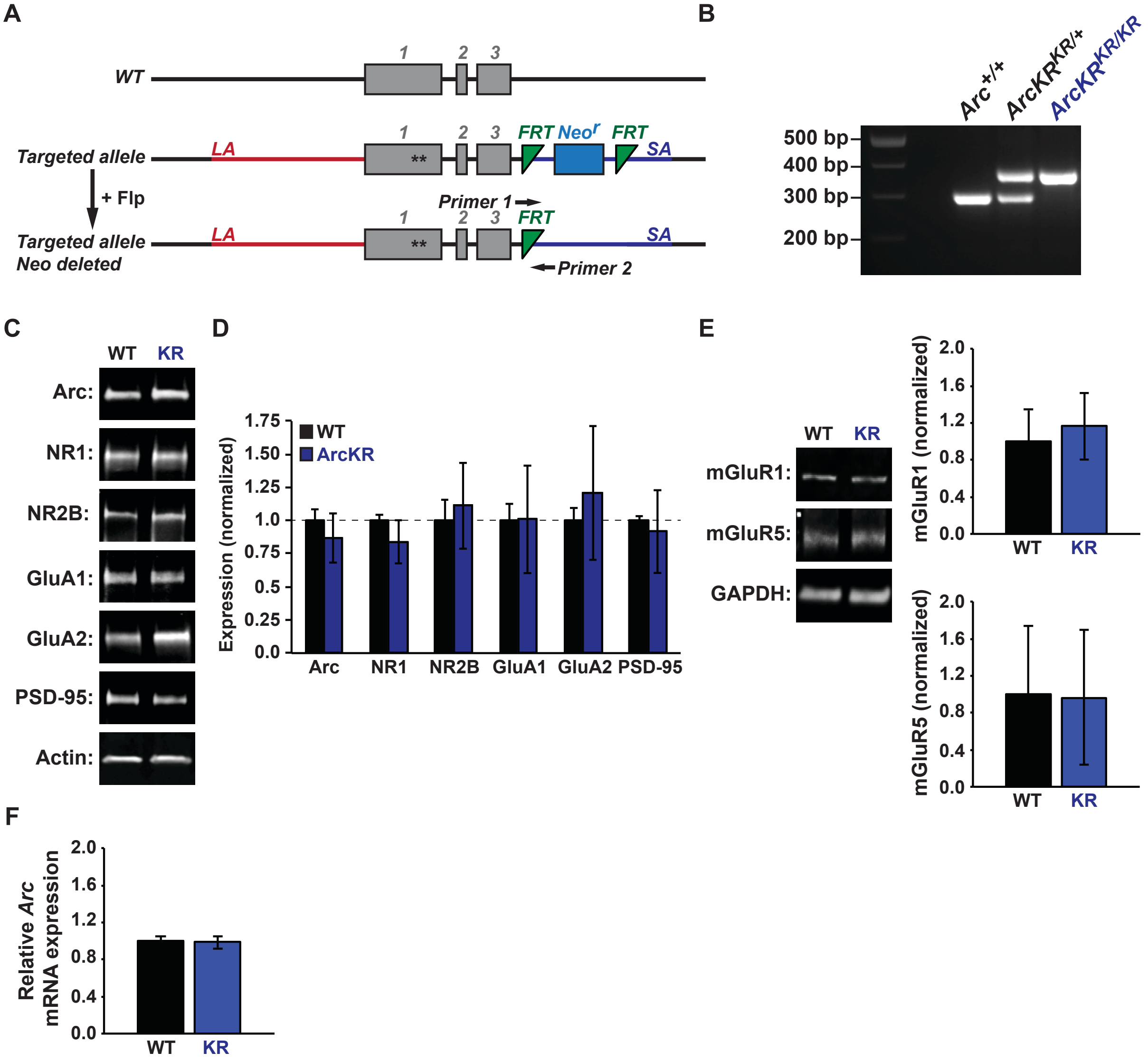
A
WT
1
2
3
Targeted allele
LA
**
FRT
Neo^r
FRT
SA
+ Flp
Primer 1
Targeted allele
Neo deleted
Primer 2
B
Arc+/+
ArcKRKR/+
ArcKRKR/KR
500 bp
400 bp
300 bp
200 bp
C
WT
KR
Arc:
NR1:
NR2B:
GluA1:
GluA2:
PSD-95:
Actin:
D
Expression (normalized)
WT
ArcKR
Arc
NR1
NR2B
GluA1
GluA2
PSD-95
E
WT
KR
mGluR1:
mGluR5:
GAPDH:
mGluR1 (normalized)
mGluR5 (normalized)
F
Relative Arc mRNA expression

Figure S2. Generation and characterization of the ArcKR mouse, Related to Figure 1

(A) Schematic of targeting strategy used to generate Arc knock-in mice. Primer positions represent sites used to distinguish wildtype (*Arc*$^{+/+}$; WT), heterozygous (*Arc*$^{KR/+}$; HET), and homozygous (*Arc*$^{KR/KR}$; ArcKR) mice. (B) Genotyping results from WT, HET, and ArcKR mice. Wildtype band size is 291 base pairs and the knock-in band size is 355 base pairs. (C) ArcKR neurons do not exhibit synaptic abnormalities. Western blot of synaptosome fractions isolated from WT or ArcKR mice. Actin was used as a loading control. (D) Quantification of synaptic protein expression in WT and ArcKR mice. All proteins are normalized to Actin. (E) Western blot of hippocampi isolated from postnatal day 60 (P60) WT or ArcKR mice. GAPDH was used as a loading control. n = 3 independent experiments. (F) Quantitative pPCR demonstrating no change in *Arc* mRNA expression between WT and ArcKR mice at P60. Hippocampi from WT and ArcKR mice were collected and the total RNA was isolated. *Arc* mRNA was determined by quantitative PCR. Data were normalized to the geometric mean of *GAPDH* reference gene and *Arc*. Each data point represents duplicate measurements from an individual mouse from 12 WT and 9 ArcKR mice. Values represent mean ± S.E.M.

Figure S3

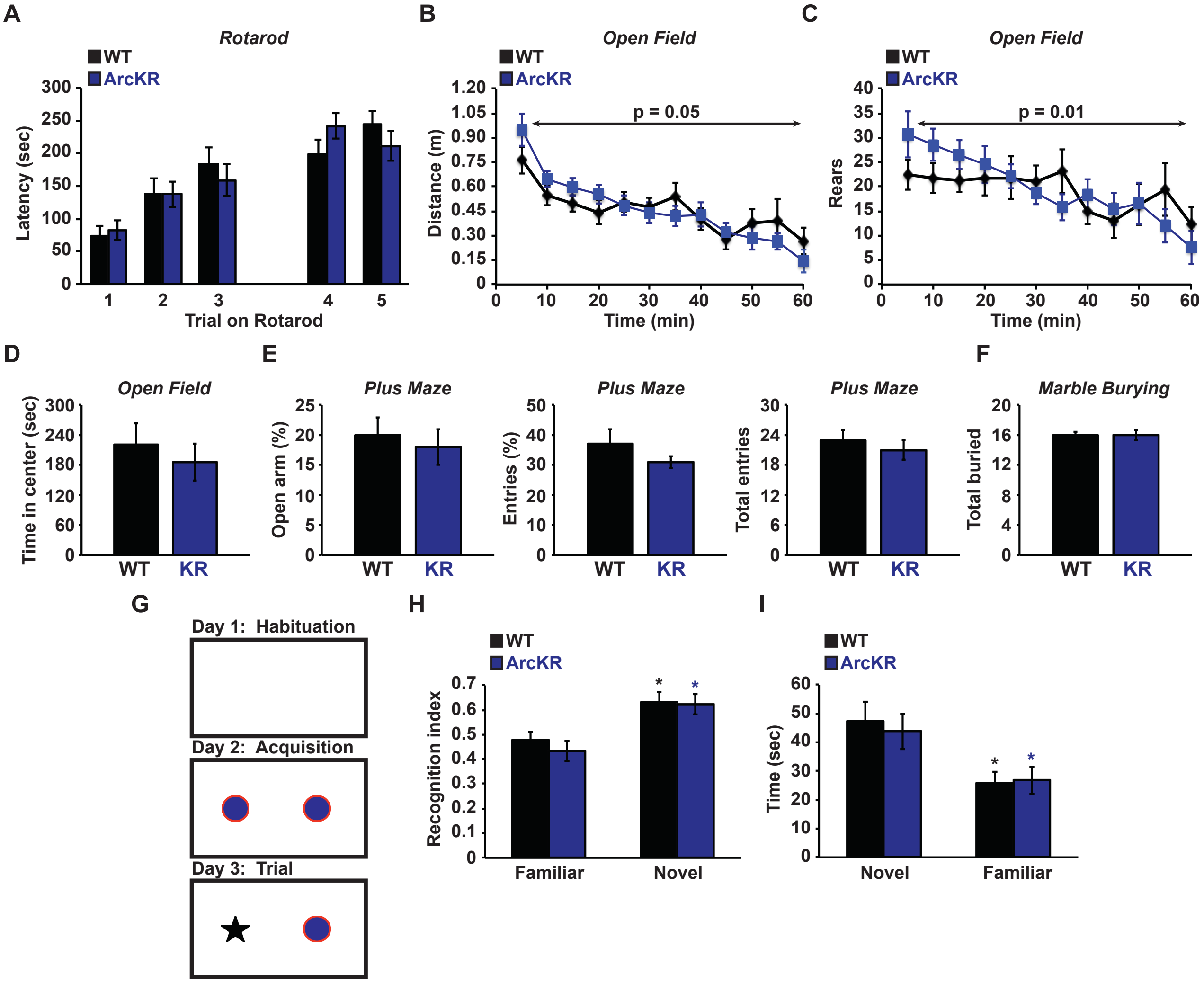
A
Rotarod
WT
ArcKR
Latency (sec)
Trial on Rotarod
B
Open Field
WT
ArcKR
Distance (m)
p = 0.05
Time (min)
C
Open Field
WT
ArcKR
Rears
p = 0.01
Time (min)
D
Open Field
Time in center (sec)
WT
KR
E
Plus Maze
Open arm (%)
Plus Maze
Entries (%)
Plus Maze
Total entries
F
Marble Burying
Total buried
G
Day 1: Habituation
Day 2: Acquisition
Day 3: Trial
H
WT
ArcKR
Recognition index
Familiar
Novel
I
WT
ArcKR
Time (sec)
Novel
Familiar

Figure S3. Behavioral characterization of ArcKR mice, Related to Figure 3

(A) Motor coordination and learning are unaltered in ArcKR mice. Graph plotting the time until mice fall off an accelerating rotarod. Mice were given three trials on day 1 (1, 2, and 3). Mice were then given 2 additional trials 48 hours later (4 and 5). (B) Initial locomotor activity in a novel open field is enhanced in ArcKR mice. Distance traveled over a time course of 60 min after placement in a novel environment. (C) Initial rearing activity in a novel open field is enhanced in ArcKR mice. Number of rears were taken within 60 min after placement in a novel environment. (D) ArcKR mice spend similar time in the center as WT mice in a novel open field. Time spent in the center of a novel open field within 60 min after placement in a novel environment. (E) ArcKR mice exhibit normal anxiety-like behavior in an elevated plus maze. Percent time spent in open arm (left), percent entries (middle), and total number of entries (right) in WT and ArcKR mice. (F) ArcKR mice exhibit normal marble burying behavior. (G) ArcKR mice have normal long-term recognition memory. Experimental setup for the Novel Object Recognition Test. (H) Novel Object Recognition Index values of WT and ArcKR mice. WT = 11 animals, ArcKR = 10 animals. (I) Total time mice spent with the Novel versus Familiar Object in the Trial phase of the NORT. Values represent mean ± S.E.M, $p \leq 0.05$. WT=12 animals and ArcKR-12 animals

Figure S4

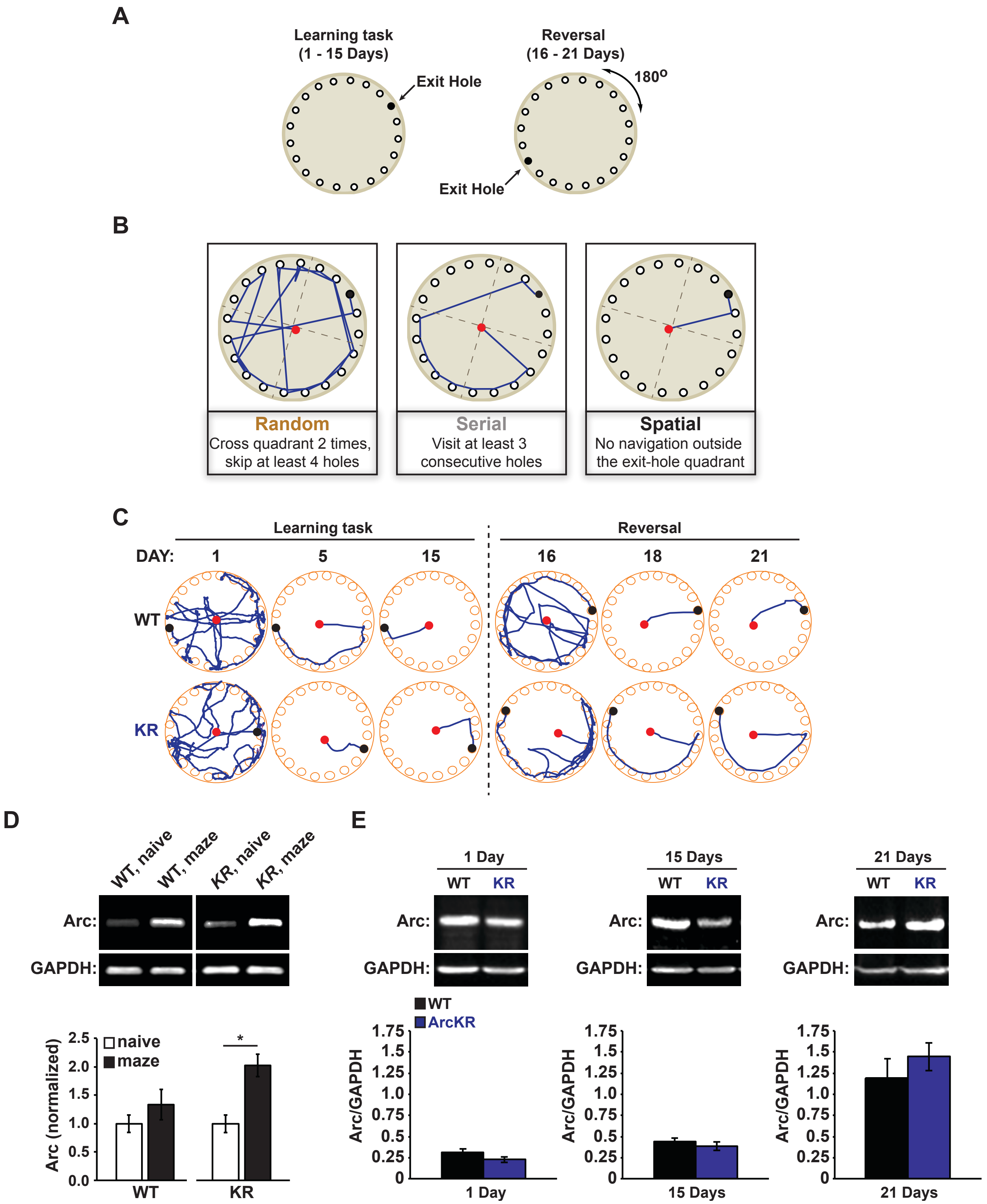
A
Learning task
(1 - 15 Days)
Exit Hole
Reversal
(16 - 21 Days)
180°
Exit Hole
B
Random
Cross quadrant 2 times, skip at least 4 holes
Serial
Visit at least 3 consecutive holes
Spatial
No navigation outside the exit-hole quadrant
C
Learning task
Reversal
DAY: 1 5 15 16 18 21
WT
KR
D
WT, naive
WT, maze
KR, naive
KR, maze
Arc:
GAPDH:
Arc (normalized)
naive
maze
*
WT
KR
E
1 Day
15 Days
21 Days
WT
KR
Arc:
GAPDH:
WT
ArcKR
Arc/GAPDH
1 Day
15 Days
21 Days

Figure S4. Arc knock-in mice exhibit deficits in cognitive flexibility, Related to Figure 3

(A) Schematic drawing showing the Barnes Maze experimental setup. Mice were tested a single time daily for 15 days. On day 16, the maze was rotated 180 degrees, mice were tested a single time daily, as before. (B) The scoring parameters used to assess learning strategies for the Barnes Maze paradigm. (C) Representative activity traces in WT and ArcKR mice during days 1, 5, 15, 16, 18, and 21. (D) *Top*, blot showing Arc protein levels obtained from hippocampal lysate of Barnes maze trained WT and ArcKR mice. Actin was used as a loading control. *Bottom*, Graph represents Arc levels normalized to Actin from D. N = 3, $p \leq 0.05$. (E) Blots showing Arc protein obtained from hippocampal lysates of Barnes maze WT and ArcKR mice trained at 1, 15, and 21 days (WT 1day: n=5; WT 15 days: n=5; WT 21 days: n = 4; ArcKR 1 day: n=6; ArcKR 15 days: n=7; ArcKR 21 days: n = 5). GAPDH was used as a loading control. Graphs represent Arc levels normalized to GAPDH. Values represent mean ± S.E.M.
