# Supplementary material for: The Temporal Dynamics of Arc Expression Regulate Cognitive Flexibility
Source: Neuron. 2018 Jun 27;98(6):1124–1132.e7. doi: 10.1016/j.neuron.2018.05.012 (PMC6030446; doi:10.1016/j.neuron.2018.05.012)
Supplement: Document S2. Article plus Supplemental Information [file mmc2.pdf]

Neuron

Report

The Temporal Dynamics of Arc Expression Regulate Cognitive Flexibility

Graphical Abstract

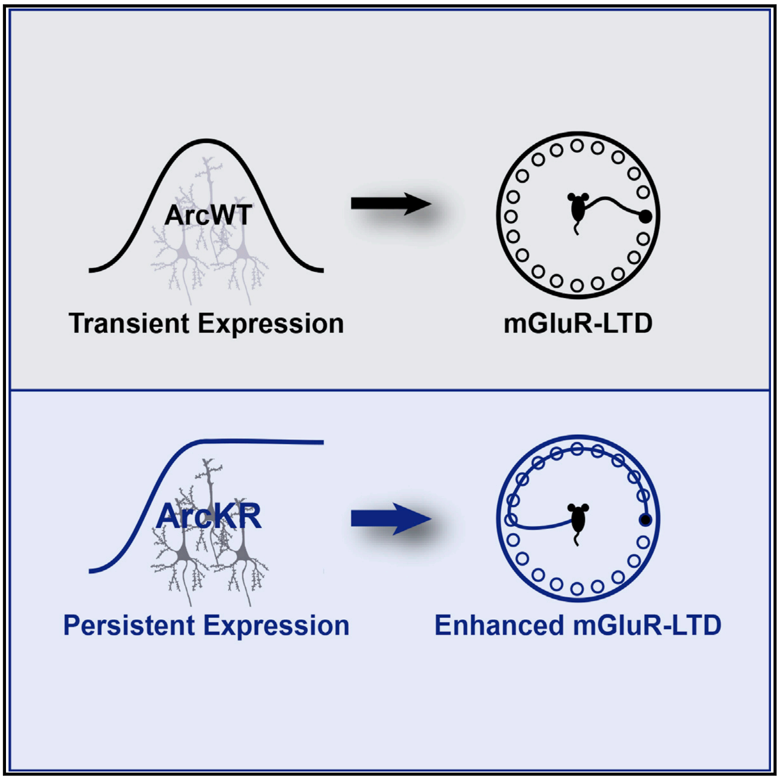

Highlights

- Mutation of Arc ubiquitination sites slows Arc degradation
- Arc knockin mice have enhanced mGluR-LTD
- The persistence of Arc reduces cognitive flexibility

Authors

Mark J. Wall, Dawn R. Collins, Samantha L. Chery, ..., Michael D. Ehlers, Angela M. Mabb, Sonia A.L. Corrêa

Correspondence

amabb@gsu.edu (A.M.M.), s.a.l.correa@bradford.ac.uk (S.A.L.C.)

In Brief

Ubiquitin-dependent proteasomal degradation of Arc tightly limits its temporal expression, yet the significance of this regulation remains unknown. We generated a mouse disrupting the temporal control of Arc removal (ArcKR). ArcKR mice exhibit enhanced mGluR-LTD and have impaired cognitive flexibility.

Wall et al., 2018, Neuron *98*, 1124–1132
June 27, 2018 © 2018 The Authors. Published by Elsevier Inc.
https://doi.org/10.1016/j.neuron.2018.05.012

CellPress

Neuron
Report

The Temporal Dynamics of Arc Expression Regulate Cognitive Flexibility

Mark J. Wall,[1] Dawn R. Collins,[2] Samantha L. Chery,[3] Zachary D. Allen,[3] Elissa D. Pastuzyn,[4] Arlene J. George,[3] Viktoriya D. Nikolova,[5] Sheryl S. Moy,[5] Benjamin D. Philpot,[6] Jason D. Shepherd,[4] Jürgen Müller,[7] Michael D. Ehlers,[8] Angela M. Mabb,[3,9,*] and Sonia A.L. Corrêa[1,7,*]
[1]School of Life Sciences, University of Warwick, Coventry, CV4 7AL, UK
[2]Warwick Medical School, University of Warwick, Coventry, CV4 7AL, UK
[3]Neuroscience Institute, Georgia State University, Atlanta, GA 30303, USA
[4]Department of Neurobiology and Anatomy, University of Utah School of Medicine, Salt Lake City, UT 84112, USA
[5]Department of Psychiatry and the Carolina Institute for Developmental Disorders, University of North Carolina, Chapel Hill, NC 27599, USA
[6]Neuroscience Center, Department of Cell Biology & Physiology, and the Carolina Institute for Developmental Disorders, University of North Carolina, Chapel Hill, NC 27599, USA
[7]Bradford School of Pharmacy and Medical Sciences, University of Bradford, Bradford, BD7 1DP, UK
[8]Biogen, Cambridge, MA 02142, USA
[9]Lead Contact
*Correspondence: amabb@gsu.edu (A.M.M.), s.a.l.correa@bradford.ac.uk (S.A.L.C.)
https://doi.org/10.1016/j.neuron.2018.05.012

SUMMARY

Neuronal activity regulates the transcription and translation of the immediate-early gene Arc/Arg3.1, a key mediator of synaptic plasticity. Proteasome-dependent degradation of Arc tightly limits its temporal expression, yet the significance of this regulation remains unknown. We disrupted the temporal control of Arc degradation by creating an Arc knockin mouse (ArcKR) where the predominant Arc ubiquitination sites were mutated. ArcKR mice had intact spatial learning but showed specific deficits in selecting an optimal strategy during reversal learning. This cognitive inflexibility was coupled to changes in Arc mRNA and protein expression resulting in a reduced threshold to induce mGluR-LTD and enhanced mGluR-LTD amplitude. These findings show that the abnormal persistence of Arc protein limits the dynamic range of Arc signaling pathways specifically during reversal learning. Our work illuminates how the precise temporal control of activity-dependent molecules, such as Arc, regulates synaptic plasticity and is crucial for cognition.

INTRODUCTION

The activity-regulated protein Arc/Arg3.1 (Arc) is essential for spatial memory acquisition and consolidation (Bramham et al., 2008; Guzowski et al., 1999; Lyford et al., 1995; Plath et al., 2006). Arc is required for protein-synthesis-dependent synaptic plasticity related to learning and memory, making it one of the key molecular players in cognition (Park et al., 2008; Shepherd and Bear, 2011; Waung et al., 2008). Arc protein expression is highly dynamic: increasing and then rapidly declining following increased network activity or exposure to a novel environment (Guzowski et al., 1999; Ramírez-Amaya et al., 2005). Retrieval of a memory also induces Arc expression which then rapidly decays (Nakayama et al., 2015; Ramírez-Amaya et al., 2005). The regulation of Arc protein induction occurs at the level of mRNA transcription, mRNA trafficking, and protein translation (Korb and Finkbeiner, 2011). Although the importance of Arc induction is clear, the role of Arc protein degradation in synaptic plasticity and learning-related behaviors is still unknown.

To determine the importance of Arc removal, we generated a mutant mouse line (ArcKR) in which ubiquitin-dependent degradation of Arc is disabled. We show that ArcKR mice display impaired cognitive flexibility that is coupled with elevated levels of Arc protein expression, a reduced threshold to induce mGluR-LTD, and enhanced mGluR-LTD. We further show that behavioral training alters *Arc* mRNA expression and modulates the magnitude of mGluR-LTD.

RESULTS

Ubiquitin-Dependent Turnover of Arc Alters AMPA Receptor Trafficking

Arc promotes AMPA receptor (AMPAR) endocytosis following activation of group I mGluRs with the agonist DHPG (Waung et al., 2008). This effect is reduced by overexpression of Triad3A/RNF216, which targets Arc for degradation. Conversely, Triad3A/RNF216 depletion increases Arc levels, thus enhancing AMPAR endocytosis (Mabb et al., 2014a). We recorded mEPSCs in neurons expressing short hairpin RNA (shRNA) directed against Triad3A/RNF216 (Figure S1A). Depletion of Triad3A/RNF216 significantly ($p = 0.026$, Figure S1A) enhanced DHPG-dependent reduction in AMPAR-mediated mEPSC amplitude

 © 2018 The Authors. Published by Elsevier Inc.
This is an open access article under the CC BY license (http://creativecommons.org/licenses/by/4.0/).

within 2–3 min compared to neurons expressing a scrambled shRNA.

Triad3A/RNF216 and Ube3A E3 ligases ubiquitinate Arc on lysines 268 and 269, targeting Arc for proteasome-mediated degradation (Greer et al., 2010; Mabb et al., 2014a). To confirm that ubiquitination of Arc modulates surface AMPAR expression, we transfected hippocampal neurons with either Arc-WT, Arc-2KR (K268/269R), or Arc-5KR (K55/136/268/269/293R) and then stained for the surface AMPAR subunit, GluA1 (Figure S1B). Overexpression of the Arc-KR mutants (Arc-2KR or Arc-5KR) had comparable effects, both producing a greater decrease in surface GluA1 expression compared to Arc-WT. Thus, a reduction in Arc protein degradation enhances GluA1 internalization (Figure S1C) and suggests that expression of a degradation-resistant Arc protein would augment AMPAR endocytosis *in vivo*.

Enhanced mGluR-LTD in the Hippocampus of ArcKR Mice

We next created an Arc knockin mouse (ArcKR) where lysine 268 and 269 were mutated to arginine to prevent Arc ubiquitination (Figures S2A and S2B). ArcKR mice were born with expected Mendelian ratios, with no differences in mortality rate or in weight of heterozygous or homozygous $Arc^{KR/KR}$ (ArcKR) mice compared to $Arc^{+/+}$ (WT) littermates. There were no significant differences in the expression of various scaffold proteins, NMDA receptors, or AMPAR subunits in synaptosomes (Figures S2C and S2D). Expression levels of mGluR1/5 and Arc protein were similar in WT and ArcKR mice as was *Arc* mRNA (Figures S2C–S2F). To confirm that proteasome-mediated turnover of Arc was impaired, we monitored Arc protein levels following addition of DHPG (100 μM, 10 min), which induces Arc translation and ubiquitination (Klein et al., 2015; Waung et al., 2008). In WT neurons, addition of DHPG increased Arc protein, peaking at 120 min post-induction and decaying near baseline levels at 480 min (Figure 1A). Addition of DHPG to ArcKR hippocampal neurons resulted in persistent Arc protein elevation (Figures 1A and 1B). To measure Arc degradation, we applied the protein synthesis inhibitor anisomycin after DHPG to halt Arc protein synthesis. In WT neurons, Arc levels were reduced after anisomycin treatment (Figure 1C), consistent with rapid Arc degradation. In contrast, this decline was significantly blunted in ArcKR neurons (Figure 1D), demonstrating the importance of proteasomal degradation in limiting the half-life of Arc protein. We have demonstrated that Arc ubiquitination via K48 linkages could be elicited by pilocarpine-induced seizures *in vivo* (Mabb et al., 2014a). Pilocarpine-induced seizures in WT mice resulted in an increase in K48-linked Arc ubiquitination, an effect that was attenuated in ArcKR mice (Figure 1E).

Consistent with the reduced surface expression of GluA1, we observed increased GluA1 endocytosis in ArcKR neurons treated with DHPG using a high-content AMPA receptor trafficking assay (Figures 1F and 1G). Intriguingly, we found a significant increase in the surface expression of the GluA2 subunit in ArcKR neurons, at short time points, indicating a potential receptor subunit replacement (Figures 1H and 1I). These findings support our previous observation that overexpression of ArcWT in hippocampal neurons increases the rectification index of AMPAR-mediated miniature-EPSC amplitude, indicating an increase in the proportion of AMPAR-containing GluA2 subunits (DaSilva et al., 2016; Wall and Corrêa, 2018).

Given the increase in GluA1-containing AMPAR endocytosis rates, we speculated that mGluR-LTD would be enhanced, as this form of plasticity requires Arc-dependent AMPAR internalization (Park et al., 2008; Waung et al., 2008). Basal synaptic transmission and synaptic plasticity were measured in hippocampal slices from ArcKR and WT littermates. ArcKR mice had unaltered basal synaptic transmission: no significant ($p > 0.05$) change in paired-pulse facilitation (Figure 2A), input-output relationship (Figure 2B), or ratio of fEPSP slope to volley amplitude (Figure 2B, inset). Thus, under basal conditions, Arc ubiquitination has little effect on synaptic AMPAR expression consistent with no changes in protein and mRNA expression (Figures S2C, S2D, and S2F). To investigate the role of Arc degradation in synaptic plasticity, we induced mGluR-LTD with DHPG (100 μM, 10 min) in hippocampal slices from ArcKR and WT littermates. We observed no significant difference between genotypes ($p = 0.29$) when DHPG was present. However, LTD was significantly enhanced in ArcKR mice (55–60 min after DHPG washout, Figures 2C and 2D). To test whether the threshold to induce LTD is reduced in ArcKR slices, we applied a lower concentration of DHPG (50 μM, 10 min). This lower concentration of DHPG was insufficient to induce LTD in WT mice but was sufficient to induce LTD in slices from ArcKR mice (Figures 2E and 2F). Thus, reduction of Arc ubiquitination reduces the threshold to induce LTD and enhances the magnitude of mGluR-LTD.

ArcKR Mice Exhibit Cognitive Inflexibility and Reduced Threshold to Induce mGluR-LTD

We considered whether ArcKR mice displayed behavioral deficits. WT and ArcKR mice had no overt motor abnormalities, similar anxiety levels (Figures S3A–S3F) and recognition memory (Figures S3G–S3I).

We next explored the role of Arc ubiquitination in hippocampal-dependent spatial learning by using a modified Barnes maze (Eales et al., 2014). Mice were tested for 21 consecutive days to test acquisition, consolidation, and expression phases of learning (days 1–15). On day 16, the platform was rotated 180°, requiring the mice to learn a new location for the exit hole (Figure S4A). No differences were observed in spatial acquisition during days 1–15 (Figures 3A and 3B). Following 180° rotation of the exit hole on day 16, there was no significant difference in distance traveled (Figure 3A), but there was a significantly higher number of errors (Figure 3B, reversal, $p = 0.02$) and selective perseverance bias (Figure 3C, $p = 0.05$) in ArcKR mice during the reversal phase (days 16–21). However, there was no difference in quadrant bias ratios during the acquisition phase (Figure 3C). This suggests that ArcKR mice show impairments in performing the task specifically during reversal learning. We next examined the strategy used to search for the maze exit hole (Figure 3D; Figure S4B). Both WT and ArcKR mice showed a similar shift from a combination of random and serial strategies to a spatial search strategy (days 1–15; Figure 3D). However, when the maze exit was rotated 180° (reversal learning), there were

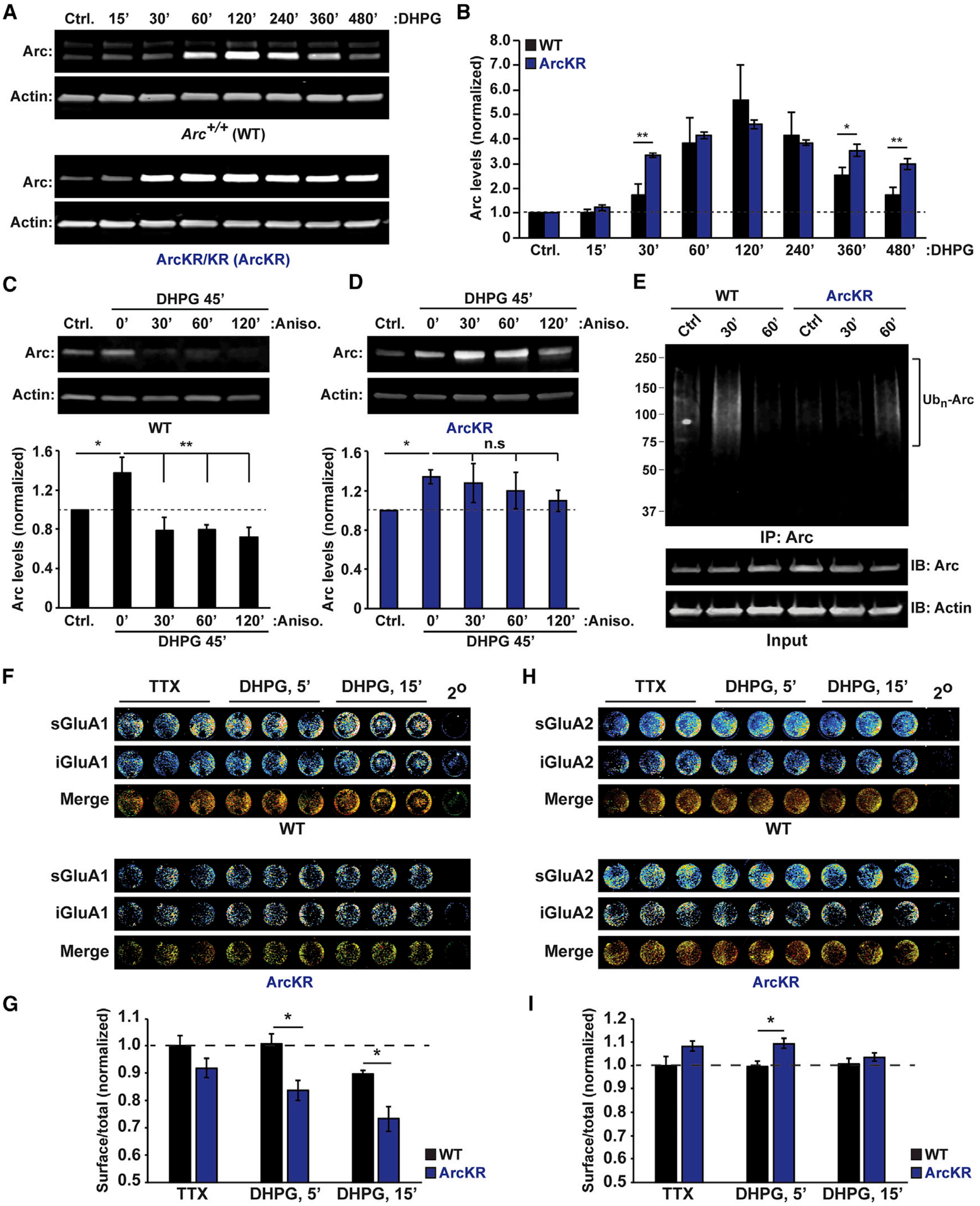

Figure 1. ArcKR Mice Exhibit Defects in Ubiquitin-Mediated Turnover of Arc

(A and B) Blots showing increased Arc protein in ArcKR hippocampal cultures treated with DHPG (100 μM DHPG; 10 min) and harvested 15, 30, 60, 120, 240, 360, and 480 min after DHPG washout.

(C) Blots showing that Arc turnover is faster in WT neurons.

(D) Blots showing that Arc turnover is blunted in ArcKR neurons.

(E) Blot of K48-linked ubiquitin showing loss of Arc ubiquitination in ArcKR mice after pilocarpine induced class III seizure. Actin was used as a loading control.

(F and G) ArcKR neurons have increased GluA1 endocytosis. Odyssey CLx scans for surface and internalized AMPAR subunits in WT and ArcKR hippocampal neurons at 5 and 15 min after DHPG washout (F). Graph represents surface fluorescence normalized to the total fluorescence intensity (G).

(H and I) The same experimental condition as in (G) showing that ArcKR neurons have increased surface levels of GluA2. Statistical comparisons were carried out using one-way ANOVA, paired and unpaired Student's t tests. *$p \leq 0.05$; **$p \leq 0.005$; n = 3 technical replicates from 3 independent experiments. Values represent mean ± SEM.

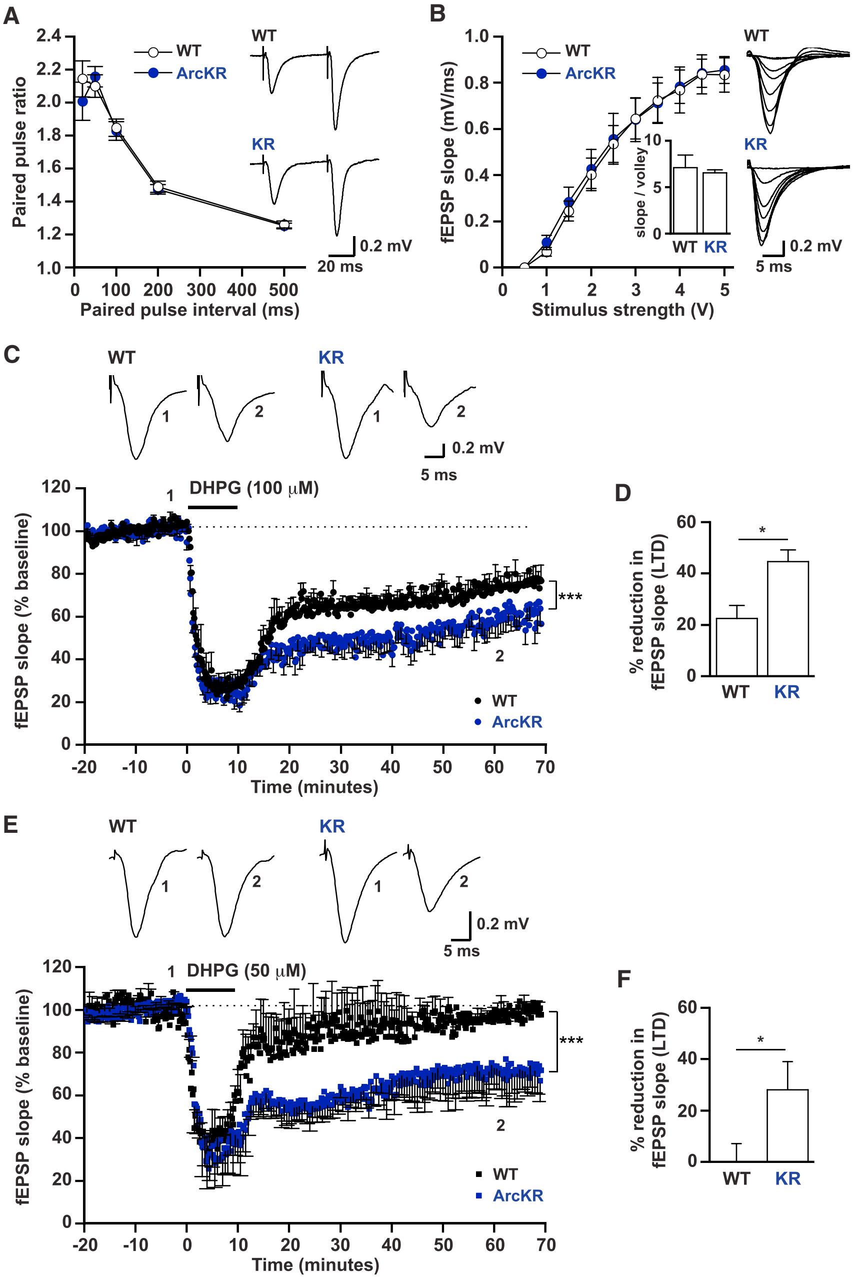

Figure 2. Defects in Arc Ubiquitination Enhance mGluR-LTD

(A) Mean paired-pulse ratio against paired-pulse interval for WT (n = 12 slices) and ArcKR mice (n = 10 slices). Inset: representative traces at an interval of 50 ms from WT and ArcKR littermates.

(B) Mean fEPSP slope against stimulus strength for WT (n = 9 slices) and ArcKR mice (n = 10 slices) with examples of superimposed averaged fEPSPs at different stimulus strengths. Inset: ratio of fEPSP slope versus volley amplitude at 40% of the stimulus strength that gives the maximum fEPSP slope (n = 6 slices per genotype).

(C) Normalized mean fEPSP slope against time for WT and ArcKR mice. After a 20 min baseline, DHPG (100 μM) was applied for 10 min and then washed out for 1 hr. Baseline fEPSP slope was analyzed at 15–20 min and LTD was analyzed at 55–60 min after DHPG application (fEPSP slope was significantly reduced following DHPG, ArcKR, $p = 0.00016$; WT, $p = 0.00013$, fEPSPs were not normalized). LTD was significantly enhanced in ArcKR slices versus WT littermates (WT: 74.3% ± 3%, n = 5 animals, 9 slices; ArcKR: 49.5% ± 4.3%, 5 mice, 12 slices, $p = 0.0004$, fEPSPs were normalized to baseline). fEPSP traces (averages of 10 fEPSPs) were taken at the times indicated by the numerals in the plot below.

(D) Mean percentage reduction in fEPSP slope (LTD) between 55 to 60 min after DHPG application ($p = 0.015$).

(E) Normalized mean fEPSP slope against time for WT and ArcKR mice. Slices were treated as in (C). In ArcKR slices, fEPSP slope was significantly reduced after 55–60 min of 50 μM DHPG application ($p = 0.0046$) but was not significantly ($p = 0.41$) reduced in WT slices (fEPSPs were not normalized). fEPSP traces (averages of 10 fEPSPs) were taken at the numerals in the lower plot.

(F) LTD was not induced in WT mice by 50 μM DHPG (reduction in slope 1.8% ± 7.7%, n = 3 mice, 5 slices) but was induced in ArcKR littermates (reduction 28% ± 11%, 3 mice, 5 slices, $p = 0.04$). Values represent mean ± SEM. Statistical comparisons were carried out with one-way ANOVA, paired and unpaired Student's t tests.

clear differences in the search strategy employed by WT and ArcKR mice. On the day of reversal, WT and ArcKR mice used similar search strategies to the first day of training (day 1) (Figures 3D and 3E; Figure S4C). However, in subsequent days, WT mice replaced random and serial for a spatial search strategy (as observed for the training before reversal). In contrast, ArcKR mice only employed a serial search strategy on days 17–19 (2–3 days following reversal), before utilizing a combination of strategies on days 20 and 21 (Figures 3D and 3E; Figure S4C). Taken together, ArcKR mice are unable to reuse strategic approaches previously acquired during task learning, suggesting cognitive inflexibility.

Reversal Learning Training Impacts on mGluR-LTD

To address whether training of a spatial-dependent task (Barnes maze) had an impact on Arc expression and subsequent hippocampal synaptic plasticity, we used hippocampi of trained WT and ArcKR littermates to either measure Arc mRNA and protein expression or to measure mGluR-LTD. Compared to WT, we observed a significantly larger decrease in the fEPSP slope during DHPG application in slices from trained ArcKR mice ($p < 0.001$, Figure 3F), and the expression of LTD was significantly increased in ArcKR slices (Figures 3F). Consistent with the enhanced decrease in the amplitude of fEPSPs during DHPG application, the levels of Arc protein were also significantly increased in hippocampal lysates obtained from ArcKR, but not from WT mice (Figure S4D). Barnes maze training of WT and ArcKR mice for 15 days resulted

CellPress

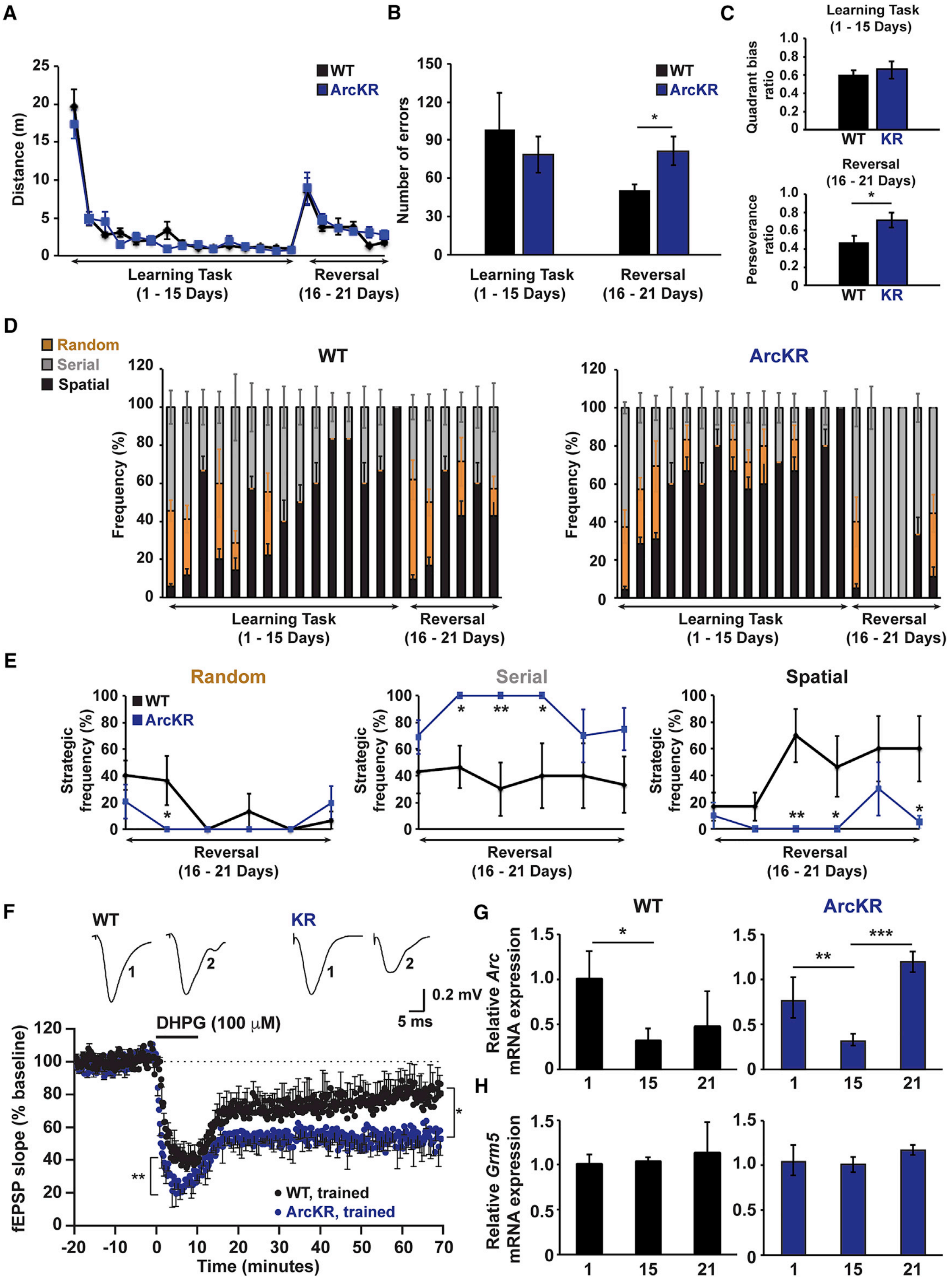

Figure 3. ArcKR Mice Have Impaired Cognitive Flexibility

(A) Distances traveled by WT and ArcKR mice during Barnes maze training.

(B) Number of errors in WT and ArcKR mice during learning (days 1–15) and reversal phase (days 16–21).

(C) Top: average quadrant bias. Bottom: perseverance ratio for WT and ArcKR mice during learning and reversal phase.

(D) Percentage time that WT and ArcKR mice used random (brown), serial (gray), and spatial (black) search strategies (n = 5 mice for WT and ArcKR).

(legend continued on next page)

in a significant reduction in *Arc* mRNA (Figure 3G) with no change in *Grm5* mRNA levels (Figure 3H) compared to the first day of training. This expression was not different between WT and ArcKR mice, indicating that *Arc* mRNA dynamics are not altered during spatial learning as has been observed in other Arc transgenic mouse models (Figure 3G) (Steward et al., 2018). Intriguingly, we found that reversal learning led to an increase in *Arc* mRNA in ArcKR mice, suggesting delayed dynamics in *Arc* mRNA induction that mirrored the learning deficits in ArcKR mice during reversal learning (Figures 3D and 3G).

To determine the effect that Barnes maze training on basal synaptic transmission and mGluR-LTD, we recorded interleaved slices from trained and naive WT and ArcKR littermates (Figure 4). Training had no effect on basal synaptic transmission in either WT or ArcKR mice (Figure 4A). However, training significantly reduced mGluR-LTD in both trained genotypes (Figures 4B and 4C). This reduction was not presynaptic (no change in paired-pulse ratios prior to and after DHPG application in trained versus naive mice, Figure 4). In ArcKR-trained slices, there was a decrease in fEPSP amplitude during DHPG application, an observation that was not seen in WT mice (Figure 4B and 4C). These findings suggest that the temporal dynamics of Arc protein expression, induced by behavioral training, modulates subsequent mGluR-LTD.

DISCUSSION

Arc protein expression is exquisitely regulated by neuronal activity. Here we have determined the functional consequences of modifying the temporal profile of Arc expression. Using a novel mouse (ArcKR) we showed: (1) mGluR1/5-dependent induction of Arc protein enhances GluA1-containing AMPAR endocytosis; (2) enhanced mGluR-LTD and a reduced threshold to induce LTD; (3) deficits in selecting strategies to perform the reversal of a spatial learned task that is coupled to the changes in LTD; and (4) increased *Arc* mRNA expression is associated with reversal learning.

Previous studies have linked alterations of mGluR-LTD to changes in spatial learning and task reversal (Eales et al., 2014; Mills et al., 2014; Xu et al., 2009). Since deficits in mGluR-LTD are associated with impairment of the acquisition/consolidation of spatial learning and poor performance with task reversal (Eales et al., 2014; Ménard and Quirion, 2012; Xu et al., 2009), it might be predicted that ArcKR mice, which show enhanced mGluR-LTD, would exhibit improved spatial learning. However, this was not the case, with ArcKR mice showing specific defects in reversal learning strategies. Instead of using a combination of random, serial, and spatial strategies, as observed in WT, ArcKR mice relied primarily on a serial strategy resulting in more errors during task reversal. These mice are unable to engage multiple strategic approaches and thus lack the cognitive flexibility required to efficiently complete the task.

How does the enhancement of LTD lead to specific cognitive deficits? During reversal learning, memories of the previously learned task are updated as new memories are acquired (McKenzie and Eichenbaum, 2011). Neural representation of such memory updating requires a precise balance between synaptic depression and potentiation (Takeuchi et al., 2013). If the amplitude of the depression is too high (i.e., enhanced LTD) or occurs too early (due to a lower threshold), this might delay or prevent the acquisition of new memories. Conversely, if LTD is impaired, this could prevent the updating of memories acquired during the acquisition phase, interfering with task reversal. Intriguingly, defects in these forms of plasticity are observed in neurological disease models, which are associated with elevated levels of Arc, (e.g., Fragile X syndrome, Angelman syndrome, and Alzheimer's disease) (Auerbach and Bear, 2010; Greer et al., 2010; Megill et al., 2015; Pastuzyn and Shepherd, 2017; Wu et al., 2011). Thus, an optimal balance of protein translation, synthesis, and turnover is required for the correct expression of mGluR-LTD and learning behavior (Citri et al., 2009; Hou et al., 2006; Klein et al., 2015).

The degree of inhibition produced by low concentrations of DHPG (50 μM) was the same in both genotypes, suggesting similar activation of mGluRs and downstream pathways, but LTD was only produced in ArcKR slices. It seems likely that Arc is degraded in WT mice and does not reach a sufficient concentration to induce LTD, whereas in ArcKR mice, Arc persists, and thus induces LTD. The amplitude of mGluR-LTD is enhanced in ArcKR mice and has a postsynaptic origin, as there were no changes in paired-pulse facilitation. This is further supported by an increased internalization of the GluA1-containing AMPAR subunit in ArcKR neurons after DHPG exposure (Figures 1F–1I). Corroborating this hypothesis is the observation that there is a significant reduction in the rectification index of AMPAR-mediated mEPSC amplitude in neurons overexpressing Arc, indicating a reduction in the number of GluA1-containing AMPAR subunits at synapses (DaSilva et al., 2016; Wall and Corrêa, 2018).

There were no changes in Arc protein or *Arc* mRNA levels between genotypes under basal conditions (Figures S2C, S2D, and S2F), consistent with the lack of differences in basal synaptic transmission (Figures 2A and 2B). Similar to previous reports (Guzowski et al., 2001), we found that prolonged behavioral

(E) Average frequency of strategy used in (D). Two-way ANOVA, post hoc Fisher's LSD, *p ≤ 0.05, **p ≤ 0.005.

(F) Normalized mean fEPSP slope against time for Barnes maze trained WT and ArcKR mice. Baseline fEPSP slope was analyzed at 15–20 min, LTD induction was analyzed at 0–5 min after DHPG and LTD expression analyzed at 55–60 min after DHPG application. Both LTD induction (**p < 0.001) and expression (WT: 78.8% ± 4.4%, n = 3, 5 slices; ArcKR: 58.1% ± 4.9%, n = 3, 7 slices, p = 0.0003) were significantly enhanced in trained ArcKR compared to WT mice. Top: representative fEPSP traces (averages of 10 fEPSPs) at the times indicated (1, 15–20 min and 2, 75–80 min).

(G) Comparison of hippocampal *Arc* mRNA after 1, 15, or 21 days of training in the Barnes maze. *Arc* mRNA was normalized to the geometric mean of 2 genes (GAPDH and GPI) and *Arc* in the mouse with the highest expression after 1 day of training was set to 1 for the WT mice. Each data point represents triplicate measurements from an individual mouse. (WT 1day: n = 5; WT 15 days: n = 5; WT 21 days: n = 4; ArcKR 1 day: n = 6; ArcKR 15 days: n = 7; ArcKR 21 days: n = 5). *p = 0.07, **p = 0.025, ***p = 0.001.

(H) *Grm5* mRNA in WT and ArcKR mice after training. Values represent mean ± SEM. Statistical comparisons were carried out with one-way ANOVA, paired and unpaired Student's t tests.

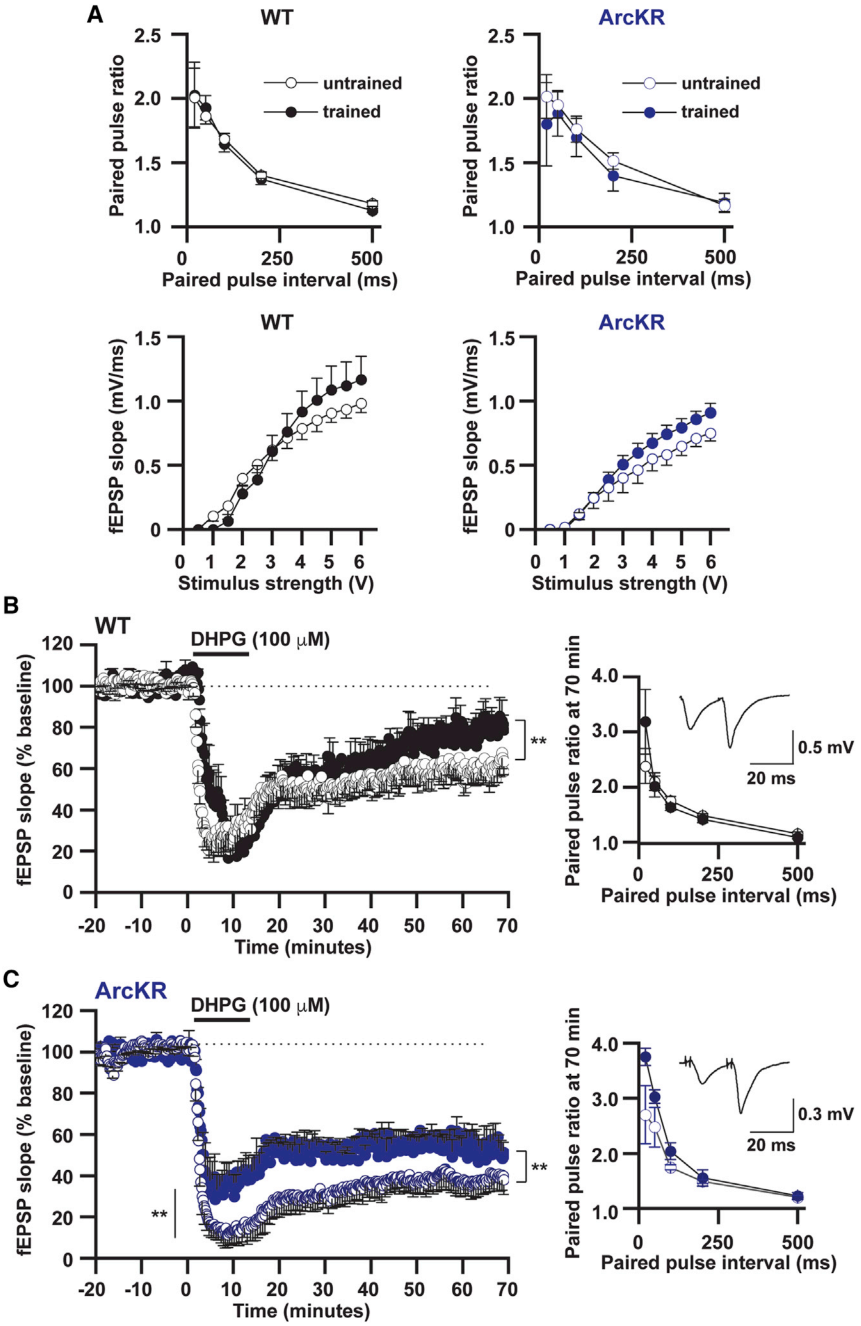

Figure 4. Reversal Learning Training Impacts on mGluR-LTD

(A) Top panels: mean paired-pulse ratio (PPR) plotted against paired pulse interval for WT (untrained n = 8 slices; trained n = 10 slices) and ArcKR mice (untrained n = 8 slices; trained n = 10 slices). Bottom panels: graphs plotting mean fEPSP slope against stimulus strength for WT (untrained n = 6 slices; trained n = 7 slices) and ArcKR mice (untrained n = 6 slices; trained n = 8 slices).

(B and C) Normalized mean fEPSP slope against time for WT (trained versus untrained) and ArcKR (trained versus untrained) mice. In WT and ArcKR mice, reversal learning significantly reduced mGluR-LTD (WT, reduction in fEPSP slope: untrained mice 39% ± 3.6%, n = 3, 7 slices; trained mice 21.3% ± 3.5%, n = 3, 6 slices, p = 0.0017; ArcKR, reduction in fEPSP slope untrained 59.9% ± 3.6%, n = 3, 7 slices; trained 47.3% ± 1.8%, n = 3, 6 slices, $p = 3.9 \times 10^{-13}$). Inset graphs: mean PPR plotted against paired-pulse interval for WT (untrained n = 8 slices; trained n = 10 slices) and ArcKR mice (untrained n = 8 slices; trained n = 10 slices) following LTD. Inset: traces at an interval of 20 ms from WT and ArcKR littermates. Values represent mean ± SEM. Statistical comparisons were carried out with a one-way ANOVA, paired and unpaired Student's t tests.

training reduced *Arc* mRNA levels (Figure 3G), although there were no changes in Arc protein expression (Figure S4E). This may reflect a fall in transcription rate, a loss of mRNA, or the slow degradation of protein. Following completion of the reversal task, there was a significant increase in *Arc* mRNA and Arc protein in ArcKR mice (Figure 3G; Figure S4E). Interestingly, the amplitude of LTD was reduced in both WT and ArcKR mice following behavioral training when compared to naive littermates (Figures 4B and 4C). This did not appear to be a consequence of changes in *Grm5* mRNA expression. The mechanism for this reduction in LTD is unclear but is likely postsynaptic, as no changes in paired pulse ratios were observed (Figures 4B and 4C). A possible explanation is that the increased expression of Arc, induced during the reversal task, partially occludes subsequent depression through a feedback mechanism that reduces induction of Arc or altered signaling pathways downstream of Arc.

Although Arc ubiquitination was attenuated in ArcKR mice (Figure 1E), there was no accumulation of Arc protein *in vivo* (Figure S4E). This suggests that this Arc ubiquitination pathway is not utilized as frequently during early stages of development. Alternatively, additional pathways might ubiquitinate Arc earlier in life and during specific phases of learning. Indeed, following stimulation of N-methyl-D-aspartate receptors, another unknown E3 ubiquitin ligase has been proposed to ubiquitinate Arc at an alternative lysine site, K136, leading to Arc degradation by the ubiquitin proteasome pathway (Gozdz et al., 2017; Mabb and Ehlers, 2018). However, this does not appear to be involved in AMPAR endocytosis as mutation of this site in addition to K268 and K269 did not further alter AMPAR internalization (Figures S1B and S1C). An alternative interpretation is that the lysosome-dependent pathway regulates Arc degradation (Nixon and Cataldo, 1995).

Recent findings suggest that Arc protein and mRNA undergo self-intercellular transfer by assembling into virus-like capsids.

This mechanism may require exosome secretion from one neuron and endocytic uptake by another (Ashley et al., 2018; Pastuzyn et al., 2018). A possible explanation for the moderate accumulation of Arc protein in ArcKR mice is that Arc ubiquitination participates in this transfer process. Evidence to support this hypothesis is highlighted by our recent findings demonstrating that the E3 ubiquitin ligase for Arc, Triad3A/RNF216 is enriched at clathrin-coated pits (CCPs), regions that participate in the endocytosis of cargo molecules. Recently, a point mutation in Arc (W197A) was shown to enhance binding to Triad3A but decrease interactions with AP-2 and dynamin (DaSilva et al., 2016). These findings suggest another functional role for Arc/Triad3A interaction. Further evidence supporting these findings is that expression of the ArcKR mutant stays longer at the plasma membrane but rarely overlaps with CCPs, suggesting that Triad3A-dependent ubiquitination might couple Arc to endocytic regions (Mabb et al., 2014a).

Our results reveal that disruption in the degradation of a single protein, Arc, is sufficient to enhance mGluR-LTD resulting in deficits in reversal learning strategy. Thus, manipulation of Arc longevity may be a strategy to restore synaptic plasticity defects in neurological disorders where Arc protein dynamics are disrupted.

STAR★METHODS

Detailed methods are provided in the online version of this paper and include the following:

- KEY RESOURCES TABLE
- CONTACT FOR REAGENT AND RESOURCE SHARING
- METHOD DETAILS
  - Animals
  - Generation of Arc knockin mice
  - Primary neuron culture and cell treatment
  - Synaptosome preparation
  - Western blotting
  - Pilocarpine-induced Arc ubiquitination assays
  - Quantitative PCR
  - Mouse behavior
  - Electrophysiology
  - Hippocampal slice preparation
  - Extracellular recording from hippocampal slices
  - Statistical Analysis

SUPPLEMENTAL INFORMATION

Supplemental Information includes four figures and can be found with this article online at https://doi.org/10.1016/j.neuron.2018.05.012.

ACKNOWLEDGMENTS

We are very grateful to T. Helton, G. deVries, E. McCoy, M. Conklin, M. Parent, and the BSU staff at the University of Warwick for their invaluable help throughout this project. This work was supported by BBSRC-BB/H018344/1 and Wellcome Trust 200646/Z/16/Z to S.A.L.C., the Whitehall Foundation (Grant 2017-05-35) and Georgia State University laboratory start-up funds to A.M.M., NIH R01NS085093 grant to B.D.P., NIH R00 NS076364 to J.D.S., S.L.C. was supported by an Institute for Maximizing Student Development (IMSD) grant, NIGMS R25GM109442.

AUTHOR CONTRIBUTIONS

Conceptualization, S.A.L.C., A.M.M., M.D.E., and J.D.S.; Validation, A.M.M. and S.A.L.C.; Methodology and Investigation, S.A.L.C., A.M.M., M.J.W., D.R.C., S.L.C., Z.D.A., E.D.P., A.J.G., V.D.N., and J.M.; Resources, S.A.L.C., A.M.M., M.D.E., J.D.S., and B.D.P.; Writing – Original Draft, S.A.L.C., A.M.M., and M.J.W.; Writing – Review & Editing, S.A.L.C., A.M.M., M.J.W., M.D.E., J.D.S., B.D.P., J.M., and S.S.M.; Visualization, A.M.M., S.A.L.C., M.J.W., J.D.S., and E.D.P.; Supervision and Project Administration, A.M.M. and S.A.L.C.; Funding Acquisition, A.M.M., S.A.L.C., B.D.P., and J.D.S.

DECLARATION OF INTERESTS

M.D.E. is an employee and shareholder of Biogen.

Received: April 4, 2017
Revised: April 6, 2018
Accepted: May 4, 2018
Published: May 31, 2018

REFERENCES

Ashley, J., Cordy, B., Lucia, D., Fradkin, L.G., Budnik, V., and Thomson, T. (2018). Retrovirus-like Gag protein Arc1 binds RNA and traffics across synaptic boutons. Cell *172*, 262–274 e211.

Auerbach, B.D., and Bear, M.F. (2010). Loss of the fragile X mental retardation protein decouples metabotropic glutamate receptor dependent priming of long-term potentiation from protein synthesis. J. Neurophysiol. *104*, 1047–1051.

Bramham, C.R., Worley, P.F., Moore, M.J., and Guzowski, J.F. (2008). The immediate early gene arc/arg3.1: regulation, mechanisms, and function. J. Neurosci. *28*, 11760–11767.

Citri, A., Soler-Llavina, G., Bhattacharyya, S., and Malenka, R.C. (2009). N-methyl-D-aspartate receptor- and metabotropic glutamate receptor-dependent long-term depression are differentially regulated by the ubiquitin-proteasome system. Eur. J. Neurosci. *30*, 1443–1450.

Corrêa, S.A., Müller, J., Collingridge, G.L., and Marrion, N.V. (2009). Rapid endocytosis provides restricted somatic expression of a K+ channel in central neurons. J. Cell Sci. *122*, 4186–4194.

DaSilva, L.L., Wall, M.J., P de Almeida, L., Wauters, S.C., Januário, Y.C., Müller, J., and Corrêa, S.A. (2016). Activity-regulated cytoskeleton-associated protein controls AMPAR endocytosis through a direct interaction with clathrin-adaptor protein 2. eNeuro *3*, 3.

Eales, K.L., Palygin, O., O'Loughlin, T., Rasooli-Nejad, S., Gaestel, M., Müller, J., Collins, D.R., Pankratov, Y., and Corrêa, S.A. (2014). The MK2/3 cascade regulates AMPAR trafficking and cognitive flexibility. Nat. Commun. *5*, 4701.

Gozdz, A., Nikolaienko, O., Urbanska, M., Cymerman, I.A., Sitkiewicz, E., Blazejczyk, M., Dadlez, M., Bramham, C.R., and Jaworski, J. (2017). GSK3α and GSK3β phosphorylate arc and regulate its degradation. Front. Mol. Neurosci. *10*, 192.

Greer, P.L., Hanayama, R., Bloodgood, B.L., Mardinly, A.R., Lipton, D.M., Flavell, S.W., Kim, T.K., Griffith, E.C., Waldon, Z., Maehr, R., et al. (2010). The Angelman syndrome protein Ube3A regulates synapse development by ubiquitinating arc. Cell *140*, 704–716.

Guzowski, J.F., McNaughton, B.L., Barnes, C.A., and Worley, P.F. (1999). Environment-specific expression of the immediate-early gene Arc in hippocampal neuronal ensembles. Nat. Neurosci. *2*, 1120–1124.

Guzowski, J.F., Setlow, B., Wagner, E.K., and McGaugh, J.L. (2001). Experience-dependent gene expression in the rat hippocampus after spatial learning: a comparison of the immediate-early genes Arc, c-fos, and zif268. J. Neurosci. *21*, 5089–5098.

Hou, L., Antion, M.D., Hu, D., Spencer, C.M., Paylor, R., and Klann, E. (2006). Dynamic translational and proteasomal regulation of fragile X mental retardation protein controls mGluR-dependent long-term depression. Neuron *51*, 441–454.

CellPress

Klein, M.E., Castillo, P.E., and Jordan, B.A. (2015). Coordination between Translation and Degradation Regulates Inducibility of mGluR-LTD. Cell Rep. *10*, 1459–1466.

Korb, E., and Finkbeiner, S. (2011). Arc in synaptic plasticity: from gene to behavior. Trends Neurosci. *34*, 591–598.

Livak, K.J., and Schmittgen, T.D. (2001). Analysis of relative gene expression data using real-time quantitative PCR and the 2(-Delta Delta C(T)) Method. Methods *25*, 402–408.

Lyford, G.L., Yamagata, K., Kaufmann, W.E., Barnes, C.A., Sanders, L.K., Copeland, N.G., Gilbert, D.J., Jenkins, N.A., Lanahan, A.A., and Worley, P.F. (1995). Arc, a growth factor and activity-regulated gene, encodes a novel cytoskeleton-associated protein that is enriched in neuronal dendrites. Neuron *14*, 433–445.

Mabb, A.M., and Ehlers, M.D. (2018). Arc ubiquitination in synaptic plasticity. Semin. Cell Dev. Biol. *77*, 10–16.

Mabb, A.M., Je, H.S., Wall, M.J., Robinson, C.G., Larsen, R.S., Qiang, Y., Corrêa, S.A., and Ehlers, M.D. (2014a). Triad3A regulates synaptic strength by ubiquitination of Arc. Neuron *82*, 1299–1316.

Mabb, A.M., Kullmann, P.H., Twomey, M.A., Miriyala, J., Philpot, B.D., and Zylka, M.J. (2014b). Topoisomerase 1 inhibition reversibly impairs synaptic function. Proc. Natl. Acad. Sci. USA *111*, 17290–17295.

McKenzie, S., and Eichenbaum, H. (2011). Consolidation and reconsolidation: two lives of memories? Neuron *71*, 224–233.

Megill, A., Tran, T., Eldred, K., Lee, N.J., Wong, P.C., Hoe, H.S., Kirkwood, A., and Lee, H.K. (2015). Defective age-dependent metaplasticity in a mouse model of Alzheimer's disease. J. Neurosci. *35*, 11346–11357.

Ménard, C., and Quirion, R. (2012). Group 1 metabotropic glutamate receptor function and its regulation of learning and memory in the aging brain. Front. Pharmacol. *3*, 182.

Mills, F., Bartlett, T.E., Dissing-Olesen, L., Wisniewska, M.B., Kuznicki, J., Macvicar, B.A., Wang, Y.T., and Bamji, S.X. (2014). Cognitive flexibility and long-term depression (LTD) are impaired following β-catenin stabilization in vivo. Proc. Natl. Acad. Sci. USA *111*, 8631–8636.

Nakayama, D., Iwata, H., Teshirogi, C., Ikegaya, Y., Matsuki, N., and Nomura, H. (2015). Long-delayed expression of the immediate early gene Arc/Arg3.1 refines neuronal circuits to perpetuate fear memory. J. Neurosci. *35*, 819–830.

Nixon, R.A., and Cataldo, A.M. (1995). The endosomal-lysosomal system of neurons: new roles. Trends Neurosci. *18*, 489–496.

Park, S., Park, J.M., Kim, S., Kim, J.A., Shepherd, J.D., Smith-Hicks, C.L., Chowdhury, S., Kaufmann, W., Kuhl, D., Ryazanov, A.G., et al. (2008). Elongation factor 2 and fragile X mental retardation protein control the dynamic translation of Arc/Arg3.1 essential for mGluR-LTD. Neuron *59*, 70–83.

Pastuzyn, E.D., and Shepherd, J.D. (2017). Activity-dependent arc expression and homeostatic synaptic plasticity are altered in neurons from a mouse model of Angelman syndrome. Front. Mol. Neurosci. *10*, 234.

Pastuzyn, E.D., Day, C.E., Kearns, R.B., Kyrke-Smith, M., Taibi, A.V., McCormick, J., Yoder, N., Belnap, D.M., Erlendsson, S., Morado, D.R., et al. (2018). The neuronal gene arc encodes a repurposed retrotransposon gag protein that mediates intercellular RNA transfer. Cell *172*, 275–288 e218.

Peixoto, R.T., Kunz, P.A., Kwon, H., Mabb, A.M., Sabatini, B.L., Philpot, B.D., and Ehlers, M.D. (2012). Transsynaptic signaling by activity-dependent cleavage of neuroligin-1. Neuron *76*, 396–409.

Plath, N., Ohana, O., Dammermann, B., Errington, M.L., Schmitz, D., Gross, C., Mao, X., Engelsberg, A., Mahlke, C., Welzl, H., et al. (2006). Arc/Arg3.1 is essential for the consolidation of synaptic plasticity and memories. Neuron *52*, 437–444.

Ramírez-Amaya, V., Vazdarjanova, A., Mikhael, D., Rosi, S., Worley, P.F., and Barnes, C.A. (2005). Spatial exploration-induced Arc mRNA and protein expression: evidence for selective, network-specific reactivation. J. Neurosci. *25*, 1761–1768.

Shepherd, J.D., and Bear, M.F. (2011). New views of Arc, a master regulator of synaptic plasticity. Nat. Neurosci. *14*, 279–284.

Shepherd, J.D., Rumbaugh, G., Wu, J., Chowdhury, S., Plath, N., Kuhl, D., Huganir, R.L., and Worley, P.F. (2006). Arc/Arg3.1 mediates homeostatic synaptic scaling of AMPA receptors. Neuron *52*, 475–484.

Steward, O., Matsudaira Yee, K., Farris, S., Pirbhoy, P.S., Worley, P., Okamura, K., Okuno, H., and Bito, H. (2018). Delayed Degradation and Impaired Dendritic Delivery of Intron-Lacking *EGFP-Arc/Arg3.1* mRNA in *EGFP-Arc* Transgenic Mice. Front. Mol. Neurosci. *10*, 435.

Takeuchi, T., Duszkiewicz, A.J., and Morris, R.G. (2013). The synaptic plasticity and memory hypothesis: encoding, storage and persistence. Philos. Trans. R. Soc. Lond. B Biol. Sci. *369*, 20130288.

Untergasser, A., Nijveen, H., Rao, X., Bisseling, T., Geurts, R., and Leunissen, J.A. (2007). Primer3Plus, an enhanced web interface to Primer3. Nucleic Acids Res. *35*, W71-4.

Wall, M.J., and Corrêa, S.A.L. (2018). The mechanistic link between Arc/Arg3.1 expression and AMPA receptor endocytosis. Semin. Cell Dev. Biol. *77*, 17–24.

Waung, M.W., Pfeiffer, B.E., Nosyreva, E.D., Ronesi, J.A., and Huber, K.M. (2008). Rapid translation of Arc/Arg3.1 selectively mediates mGluR-dependent LTD through persistent increases in AMPAR endocytosis rate. Neuron *59*, 84–97.

Wu, J., Petralia, R.S., Kurushima, H., Patel, H., Jung, M.Y., Volk, L., Chowdhury, S., Shepherd, J.D., Dehoff, M., Li, Y., et al. (2011). Arc/Arg3.1 regulates an endosomal pathway essential for activity-dependent β-amyloid generation. Cell *147*, 615–628.

Xu, J., Zhu, Y., Contractor, A., and Heinemann, S.F. (2009). mGluR5 has a critical role in inhibitory learning. J. Neurosci. *29*, 3676–3684.

STAR★METHODS

KEY RESOURCES TABLE

REAGENT or RESOURCE	SOURCE	IDENTIFIER
Antibodies		
Rabbit polyclonal anti-Arc/Arg3.1	Synaptic Systems	Cat#156 003, RRID: AB_887694
Mouse monoclonal (6C5) anti-GAPDH	Abcam	Cat#ab8245, RRID: AB_2107448
Mouse monoclonal (MAB374) anti-GAPDH	Millipore	Cat#MAB374, RRID: AB_2107445
Mouse monoclonal (RH95) anti-GluA1 N-terminal	EMD Millipore	Cat#MAB2263MI, RRID: AB_11212678
Mouse monoclonal (6C4) anti-GluA2	ThermoFisher Scientific	Cat#32-0300, RRID: AB_2533058
Rabbit polyclonal anti-mGluR1A	Sigma	Cat#G9665, RRID:AB_259995
Rabbit polyclonal anti-mGluR5	EMD Millipore	Cat#AB5675, RRID:AB_2295173
Goat polyclonal anti-NR1	Santa Cruz Biotechnology	Cat#sc-1467, RRID: AB_670215
Goat polyclonal anti-NR2B	Santa Cruz Biotechnology	Cat#sc-1469, RRID: AB_670229
Rabbit polyclonal anti-PSD95	Synaptic Systems	Cat#124 002, RRID: AB_887760
Mouse monoclonal (GT5512) anti-β-Actin	Genetex	Cat#GTX629630, RRID: AB_2728646
Donkey anti-mouse Alexa Fluor 555	Thermo Fisher Scientific	Cat#A31570, RRID AB_2536180
Donkey anti-rabbit Alexa Fluor 488	Jackson ImmunoResearch	Cat#715-545-150, RRID: AB_2340846
IRDye 680RD Goat anti-Mouse IgG (H+L)	Li-COR Biosciences	Cat# 926-68070, RRID: AB_10956588
IRDye 800CW Goat anti-Rabbit IgG (H+L)	Li-COR Biosciences	Cat# 926-32211, RRID: AB_621843
IRDye 800CW Donkey anti-Mouse IgG (H+L)	Li-COR Biosciences	Cat# 926-32212, RRID: AB_621847
Goat anti-rabbit IgG HRP-linked antibody	Cell Signaling Technology	Cat#7074, RRID: AB_2099233
Goat anti-mouse IgG HRP-linked antibody	Jackson Immuno- Research	Cat#115-035-174, RRID: AB_2338512
Chemicals, Peptides, and Recombinant Proteins		
Papain	Worthington Biochemicals	Cat#LS003126
Deoxyribonuclease I from bovine pancreas	Sigma-Aldrich	Cat# D4513
Poly-L-lysine	Sigma-Aldrich	Cat#P2636-100MG
cytosine β-D-arabinofuranoside	Sigma-Aldrich	Cat# C1768-100MG
Anisomycin	Sigma-Aldrich	Cat#A9789, CAS 22862-76-6
Fluoromount aqueous mounting medium	Sigma-Aldrich	Cat#F4680-25ML
cOmplete, Mini, EDTA-free Protease Inhibitor Cocktail	Sigma-Aldrich	Cat#11836170001
Phosphatase Inhibitor Cocktail 2	Sigma-Aldrich	Cat#P5726
Picrotoxin	Tocris Bio-Techne	Cat#1128, CAS 124-87-8
L689,560	Tocris Bio-Techne	Cat# 0742/10, CAS 139051-78-8
(RS)-3,5-DHPG	Tocris Bio-Techne	Cat#0342, CAS 19641-83-9
Tetrodotoxin citrate	Tocris Bio-Techne	Cat#1069, CAS 18660-81-6
RNAlater	QIAGEN	Cat#76104
QIAshredder	QIAGEN	Cat#79654
RNase-Free DNase Set	QIAGEN	Cat#79254
TRIzol	ThermoFisher Scientific	Cat#12044977
Normal donkey serum	Jackson ImmunoResearch	Cat#017-000-121
Odyssey Blocking Buffer (TBS)	Li-COR	Cat#927-50003
Saponin	ACROS Organics	Cat# 419231000, CAS 74499-23-3
Critical Commercial Assays		
RNeasy Lipid Tissue Mini Kit	QIAGEN	Cat#74804
Transcriptor First Strand cDNA Synthesis Kit	Roche	Cat#04379012001
PowerUp SYBR Green Master Mix	ThermoFisher Scientific	Cat#15350929

(*Continued on next page*)

Continued

REAGENT or RESOURCE	SOURCE	IDENTIFIER
ECL Supersignal West Pico	ThermoFisher Scientific	Cat#34080
BCA Protein Assay kit	ThermoFisher Scientific	Cat#23225
Lipofectamine 2000 Transfection Reagent	ThermoFisher Scientific	Cat#11668027
Oligonucleotides		
ARC-KI-NDEL1: cttattggagtatgtgccatttctc	This paper, genotyping	N/A
ARC-KI-NDEL2: cattgaccctgtctccagattc	This paper, genotyping	N/A
Arc-L: tgttgaccgaagtgtccaag	This paper, qPCR	N/A
Arc-R: aagttgttctccagcttgcc	This paper, qPCR	N/A
mGluR5-L: cagtccgtgagcagtatgg	This paper, qPCR	N/A
mGluR5-R: gcccaatgactcccacta	This paper, qPCR	N/A
GAPDH-L: ggcaaattcaacggcacagt	This paper, qPCR	N/A
GAPDH-R: gggtctcgctcctggaagat	This paper, qPCR	N/A
mGPI-L: agctgcgcgaactttttgag	This paper, qPCR	N/A
mGPI-R: tatgcccatggttggtgttg	This paper, qPCR	N/A
Recombinant DNA		
prK5-myc-Arc	Mabb et al., 2014a	N/A
prK5-myc-ArcKR	Mabb et al., 2014a	N/A
prK5-myc-Arc-5KR	Mabb et al., 2014a	N/A
pLentilox 3.7 LL-Scramble control shRNA	Mabb et al., 2014a	N/A
pLentilox 3.7 LL-*Triad3/RNF216*-shRNA #2	Mabb et al., 2014a	N/A
Software and Algorithms		
Primer3Plus	Untergasser et al., 2007	http://www.bioinformatics.nl/cgi-bin/primer3plus/primer3plus.cgi
StepOne Plus	Applied Biosystems	N/A
SPSS 22	IBM	N/A
Any-MAZE	Stoelting	https://www.stoeltingco.com; RRID: SCR_014289
Origin	Microcal	https://www.originlab.com/; RRID: SCR_002815
Multiclamp 700B	Molecular Devices	N/A
Digidata 1440A	Molecular Devices	N/A
pClamp 10	Molecular Devices	RRID: SCR_011323
MiniAnalysis Program	SynaptoSoft	http://www.synaptosoft.com/MiniAnalysis; RRID: SCR_002184
Spike 2 Vs 7.08	Micro CED	http://ced.co.uk/products/spkovin; RRID: SCR_000903
ImageJ	NIH	https://imagej.nih.gov/ij/; RRID: SCR_003070
FIJI (Fiji is Just ImageJ)	NIH	http://fiji.sc/; RRID: SCR_002285

CONTACT FOR REAGENT AND RESOURCE SHARING

As Lead Contact, Angela M. Mabb is responsible for all reagent and resource requests. Please contact Angela M. Mabb at amabb@gsu.edu with requests and inquiries.

METHOD DETAILS

Animals

Mice were kept in standard housing with littermates, provided with food and water *ad libitum* and maintained on a 12:12 (light-dark) cycle. All behavioral tests were conducted in accordance with the *National Institutes of Health Guidelines for the Use of Animals*. All behavioral studies with the exception of the Barnes Maze test were conducted using approved protocols at the University of North Carolina, Chapel Hill. The Barnes Maze test and the hippocampal slice experiments were performed at the University of Warwick. The

mice were treated in accordance with the Animal Welfare and Ethics Committee (AWERB) and experiments were performed under the appropriated project licenses with local and national ethical approval. Samples sizes for behavioral and slice experiments were calculated using variance from previous experiments to indicate power, which statistical analysis significance was set at 95%. Primary neuron culture, pilocarpine seizure experiments, and isolation of brain tissue for biochemical experiments were approved by the Georgia State University Institutional Animal Care and Use Committee.

Generation of Arc knockin mice

Arc knockin mice were produced by the ingenious targeting laboratory (Ronkonkoma, NY). Gene targeting was performed in iTL IC1 (C57BL/6) ES cells to introduce 2 point mutations within Exon 1 of the *Arc* gene. When encoded, the introduction of these point mutations leads to a substitution of Lysine to Arginine in positions 268 and 269, sites that were previously identified as being ubiquitinated by Triad3A and Ube3a (Greer et al., 2010; Mabb et al., 2014a). ES cells were screened and positive clones were microinjected into BALB/c blastocysts and transferred to pseudopregnant female mice. Resulting chimeras with a high percentage black coat color were mated to C57BL/6 FLP mice to remove the Neo cassette, and backcrossed five times to C57/BL6 mice. $Arc^{+/+}$ (WT) were distinguished from $Arc^{KR/KR}$ (ArcKR) homozygous mice by genotyping for the presence of the one remaining FRT site after Neo deletion using the following primer sets: ARC-KI-NDEL1: 5′-cttattggagtatgtgccatttctc-3′ (Primer 1) and ARC-KI-NDEL2: 5′-cattgaccctgtctc cagattc-3′ (Primer 2) where the wild-type band size is 291 base pairs and the knockin band size is 355 base pairs.

Primary neuron culture and cell treatment

Primary hippocampal neurons of mixed sex were isolated from P0-1 mice as previously described (Corrêa et al., 2009). To assess Arc levels following mGluR-LTD, DIV12 - 14 primary hippocampal neurons were pre-treated with 2 μM TTX (Tocris) for 4 hr. TTX was washed out and 100 μm (*S*)-3,5-dihydroxyphenylglycine (DHPG, Tocris) was applied for a total of 10 min. Cells were harvested 45 min later following DHPG washout. To block protein synthesis of Arc, 20 μM anisomycin (Sigma) was added at the 45 min time point and cells were harvested 30, 60, and 120 min later.

The primary cortical neuron culture protocol was based on (Shepherd et al., 2006). Cortices of mixed sex were dissected from E18 rat embryos. Cortices were dissociated in DNase (0.01%; Sigma-Aldrich) and papain (0.067%; Worthington Biochemicals), then triturated with a fire-polished glass pipette to obtain a single-cell suspension. Cells were pelleted at 1000x*g* for 4 min, the supernatant removed, and cells resuspended and counted with a TC-20 cell counter (Bio-Rad). Neurons were plated on glass coverslips (Carolina Biological Supply, Burlington, NC) coated with poly-l-lysine (0.2 mg/mL; Sigma-Aldrich) in 12-well plates (Greiner Bio-One) at 100,000 cells/mL. Neurons were initially plated in Neurobasal media containing 5% horse serum, 2% GlutaMAX, 2% B-27, and 1% penicillin/streptomycin (Thermo Fisher Scientific) in a 37°C incubator with 5% CO_2. On DIV4, neurons were fed via half media exchange with Neurobasal media containing 1% horse serum, GlutaMAX, and penicillin/streptomycin, 2% B-27, and 5 μM cytosine β-d-arabinofuranoside (AraC; Sigma-Aldrich). Neurons were fed every three days thereafter.

Neuron transfection

At DIV14, transfections were performed using Lipofectamine 2000 (Thermo Fisher Scientific) as described previously (Shepherd et al., 2006). Immunostaining was performed 16 hr later.

Immunocytochemistry

At DIV15, transfected neurons were live-labeled for surface GluA1 receptors (Shepherd et al., 2006). Neurons were washed twice at 10°C with 4% sucrose/1X phosphate-buffered saline (PBS; 10X: 1.4 M NaCl, 26.8 mM KCl, 62 mM Na_2HPO_4, 35.3 mM KH_2PO_4, pH 7.4), then incubated in anti-GluA1-NT diluted in MEM containing 2% GlutaMAX, 2% B-27, 15 mM HEPES (Thermo Fisher Scientific), 1 mM sodium pyruvate (Thermo Fisher Scientific), and 33 mM glucose at 10°C for 20 min. Neurons were then fixed for 15 min with 4% sucrose/4% paraformaldehyde (Thermo Fisher Scientific) in 1X PBS, then incubated in Alexa Fluor 555 (Thermo Fisher Scientific) to label only surface GluA1. Following this, neurons were permeabilized for 10 min with 0.2% Triton X-100 (Amresco) in 1X PBS, and blocked for 30 min in 5% normal donkey serum (Jackson ImmunoResearch) in 1X PBS. Neurons were then incubated with rabbit anti-Arc antibody (Synaptic Systems), diluted in block for 1 hr at RT, washed 3 × 5 min in 1X PBS, and incubated in secondary antibody (Alexa Fluor 488; Jackson ImmunoResearch) diluted in block for 1 hr at RT. Neurons on coverslips were mounted on glass slides in Fluoromount (Thermo Fisher Scientific) and dried overnight at RT.

Neuron imaging and analysis

A total of 15 transfected and untransfected neurons were imaged at 60X on an Olympus FV1000 confocal microscope (Tokyo, Japan). GluA1 immunostaining was analyzed using ImageJ software. The most intense immunostaining was used to set an arbitrary pixel intensity threshold, which was applied to every image in the experiment. Integrated density of each puncta in two 25-μm dendrite segments/neuron was measured and summed to obtain a total integrated density of the puncta on the dendritic segment.

High-content AMPA receptor trafficking assay

Primary hippocampal neurons were prepared as stated above and plated in 96-well microplates (Molecular Probes) at a density of 20,000 cells per well. Neurons were fed as previously described (Corrêa et al., 2009). At DIV 14, neurons were treated with 2 μM TTX (Tocris) for 4 hr. Following treatment, neurons were cooled to room temperature and incubated with a 1:150 dilution of anti-GluA1 (Millipore, MAB2263MI) or anti-GluA2 (Invitrogen, Clone 6C4) antibody prepared in conditioned media. Neurons were incubated for 20 min at room temperature to allow antibody binding. Samples were washed 3 times with room temperature Neurobasal medium (GIBCO) and then treated with vehicle or 100 μm DHPG for 10 min. After 10 min, DHPG was washed out and neurons were fixed with

4% paraformaldehyde/4% sucrose in PBS for 20 min at 4°C. Neurons were then washed with 1X DPBS (GIBCO) and blocked in Odyssey Blocking Buffer (Li-COR) for 90 min at room temperature. To measure surface receptors, neurons were incubated for 1 hr in a 1:1,500 dilution of IRDye 680RD Goat anti-Mouse IgG (H+L) (Li-COR) secondary antibody. Neurons were then washed with TBS (50 mM Tris-HCl, 150 mM NaCl, pH7.6) 5 times and then fixed with 4% paraformaldehyde/4% sucrose in PBS for 20 min at 4°C. Neurons were then washed with TBS 2 more times and permeabilized in TBS containing 0.2% saponin (ACROS Organics) for 15 min at room temperature. Neurons were then blocked in Odyssey Blocking Buffer for 90 min at room temperature. To label the internalized pool of receptors, neurons were incubated for 1 hr in a 1:1,500 dilution of IRDye 800CW Donkey anti-Mouse IgG (H+L) (Li-COR) secondary antibody. Cells were then washed with TBS 5 times and imaged on the Odyssey Clx Imaging System (LI-COR) with a resolution of 84 μm, medium quality and a 3 mm focus offset. Images were processed in FIJI where ROIs were drawn on each well. The integrated density was measured on ROIs superimposed on the 680 (surface receptor pool) and 800 (internal receptor pool) channels individually. Experiments were run in triplicate and integrated density values for each channel in individual experimental wells were subtracted to a secondary only control. To calculate changes in surface receptor expression, the following calculation was used: R_S/R_T, where R_S represents the integrated density of surface receptors and R_T represents the integrated density of surface receptors + integrated density of internal receptors.

Synaptosome preparation

Synaptosomes were prepared as previously described (Mabb et al., 2014a). Briefly, hippocampi were dissected from male and female P21 WT and ArcKR mice. Hippocampi were lysed in 10 volumes of HEPES-buffered sucrose (0.32 M sucrose, 4 mM HEPES pH7.4 containing protease and phosphatase inhibitors (Roche) and homogenized using a motor driven glass-teflon homogenizer at ~900 rpm (10-15 strokes). The homogenate was centrifuged at 800-1000 x g at 4°C to remove the pelleted nuclear fraction. The resultant supernatant was spun at 10,000 x g for 15 min to yield the crude synaptosomal pellet. The pellet was resuspended in 10 volumes of HEPES-buffered sucrose and then respun at 10,000 x g for another 15 min to yield the crude synaptosomal fraction. The resulting pellet was lysed by hypoosmotic shock in 9 volumes ice cold H_20 plus protease/phosphatase inhibitors (Roche) and three strokes of a glass-teflon homogenizer and rapidly adjusted to 4 mM HEPES using 1 M HEPES, pH 7.4 stock solution. Samples were mixed constantly at 4°C for 30 min to ensure complete lysis. The lysate was centrifuged at 25,000 x g for 20 min to yield a supernatant (crude synaptic vesicle fraction) and a pellet (lysed synaptosomal membrane fraction). The pellet was resuspended in HEPES-buffered sucrose and used for western analysis.

Western blotting

Hippocampal lysate obtained from WT and ArcKR mice was prepared as previously described (Eales et al., 2014). Western blots were performed as previously described (Eales et al., 2014; Mabb et al., 2014b). Membranes were probed with the following antibodies: rabbit anti-Arc (Synaptic Systems, 1:1,000), goat anti-NR1 (Santa Cruz Biotechnology, 1:1,000), goat anti-NR2B (Santa Cruz Biotechnology, 1:1,000), mouse anti-GluA1 (EMD Millipore, Clone RH95, 1:500), mouse anti-GluA2 (Invitrogen, Clone 6C4, 1:500), rabbit anti-mGluR1A (Sigma, G9665, 1:500), rabbit anti-mGluR5 (EMDMillipore, AB5675, 1:500), rabbit anti-PSD-95 (Synaptic Systems, 1:1,000), mouse anti-β-Actin (Genetex, 1:5,000), mouse anti-GAPDH (Millipore, MAB374, 1:3,000 or Abcam, ab8245, 1:5,000). The following secondary antibodies were used: IRDye 680RD Goat anti-Mouse IgG (H+L) (Li-COR, 1:20,000), IRDye 800CW Goat anti-Rabbit IgG (H+L) (Li-COR, 1:15,000), IRDye 800CW Donkey anti-Goat IgG (H+L) (LI-COR, 1:15,000), Goat anti-Rabbit IgG-HRP H^+L (Cell Signaling, 1:10,000) and Goat anti-Mouse IgG HRP LC (Jackson ImmunoResearch, 1:20,000). Blots were imaged using the Odyssey Clx Imaging System (LI-COR) or the ChemiDoc MP Imaging System (Bio-Rad).

Pilocarpine-induced Arc ubiquitination assays

Pilocarpine seizures were induced in postnatal day 60–70 male and female WT and ArcKR mice as previously described (Peixoto et al., 2012). Hippocampi from mice were harvested 30 min after the presence of Class III seizure onset. Arc protein ubiquitination was measured as previously described (Mabb et al., 2014a).

Quantitative PCR

RNA extraction and cDNA synthesis

Hippocampi from trained and naive WT and ArcKR mice were collected, submerged in RNAlater, and stored at −20°C until processed. The tissue was transferred into TRIzol reagent (Fisher Scientific), disrupted using sterile pestles and homogenized by passage through a QIAshredder column (QIAGEN). The homogenization was followed by chloroform phase separation and purification of the total RNA using the RNeasy Lipid Tissue Mini Kit (QIAGEN). Purified RNA was subjected to on-column DNase treatment (Fisher Scientific) and the concentration and purity of the RNA was assessed spectrophotometrically using the NanoDrop ND-1000 (NanoDrop). RNA used had an A_{260}/A_{280} ratio of 1.9–2.25. First-strand cDNA synthesis was performed using the Transcriptor First Strand cDNA Synthesis Kit (Roche) using an anchored oligo$(dT)_{18}$ primer, according to the manufacturer's protocol.

qPCR

Primers were designed with the help of Primer3Plus software (Untergasser et al., 2007). qPCR was performed using a StepOnePlus Real-Time PCR System (Applied Biosystems, Life Technologies). Each reaction comprised of 2 μL of diluted cDNA, 5 μL PowerUp SYBR Green Master Mix (ThermoFisher) and 500 nM primers in a final volume of 10 μL. The PCR cycling conditions were as

follows: 50°C for 2 min, 95°C for 2 min, then 40 cycles of 95°C for 15 s and 60°C for 1 min. Cycling was followed by melt curve recording between 60°C and 95°C. Primer standard curves were performed to estimate the PCR efficiencies for each primer pair. Cycle threshold (Ct) values were determined by the StepOne Plus software and adjusted manually. All qPCR reactions were run in duplicate (for analysis of P60 mice) or triplicate (for analysis of Barnes maze trained mice). A mean C_t value was calculated for each primer pair and each experimental condition. Relative quantification of *Arc* and *Grm5* mRNA was performed using the $2^{-\Delta\Delta Ct}$ method (Livak and Schmittgen, 2001). Data were normalized to the geometric mean of GAPDH and/or GPI (Glucose-6-Phosphate Isomerase) and presented as expression relative to a standard condition as indicated in the figure legends. Primer sequences are as follows: Arc-L tgttgaccgaagtgtccaag; Arc-R aagttgttctccagcttgcc; mGluR5-L cagtccgtgagcagtatgg; mGluR5-R gcccaatgactc ccacta; GAPDH-L ggcaaattcaacggcacagt; GAPDH-R gggtctcgctcctggaagat; mGPI-L agctgcgcgaactttttgag; mGPI-R tatgcccatgg ttggtgttg.

Mouse behavior

Behavior cohort

For behavior experiments, 12 WT and 12 ArcKR mice were used. Mice were sex balanced and housed separately by sex in groups of 4 (2 animals per genotype). No deaths occurred throughout the course of all behavioral studies. All behavioral tests were performed with the experimenter blinded to genotype.

Rotarod

8-week-old mice were tested for motor coordination and learning on an accelerating rotarod (Ugo Basile, Stoelting). For the first test session, mice were given three trials, with 45 s between each trial. Two additional trials were given 48 hr later. Rpm (revolutions per min) was set at an initial value of 3, with a progressive increase to a maximum of 30 rpm across 5 min (the maximum trial length). The latency to fall from the top of the rotating barrel was recorded.

Open field test

Exploratory activity in a novel environment was assessed in 8-week-old mice by a one-hour trial in an open field chamber (41 cm x 41 cm x 30 cm) crossed by a grid of photobeams (VersaMax system, AccuScan Instruments). Counts were taken of the number of photobeams broken during the trial in five-min intervals, with separate measures for locomotion (total distance traveled) and rearing movements. Time spent in the center region of the open field was measured as an index of anxiety-like behavior.

Elevated plus maze

The elevated plus maze was used to assess anxiety–like behavior, based on a natural tendency of mice to actively explore a new environment, versus a fear of being in an open area. Mice (7-week-old) were given one five-min trial on the plus maze, which had two walled arms (the closed arms, 20 cm in height) and two open arms. The maze was elevated 50 cm from the floor, and the arms were 30 cm long. Mice were placed on the center section (8 cm x 8 cm) and allowed to freely explore the maze. Measures were taken of time spent in, and number of entries into, the open and closed arms of the maze.

Marble-burying assay

To measure anxiety-like behaviors, 11-week-old mice were placed in a Plexiglas cage located in a sound-attenuating chamber with ceiling light and fan. The cage contained 5 cm of corncob bedding, with 20 black glass marbles (14 mm diameter) arranged in an equidistant 5 X 4 grid on top of the bedding. Subjects were given access to the marbles for 30 min. The number of marbles buried (defined by two thirds of the marble being covered by the bedding) was measured.

Novel object recognition test

Mice (22-week-old) were habituated in a Plexiglas cage containing 2 cm of corncob bedding for 30 min. 24 hr later (Acquisition phase), two of the same objects were placed in the same habituated cage containing 2 cm of corncob bedding. Mice were allowed to explore both objects for a total of 30 min. 24 hr later (Trial phase), one of the objects was replaced with a novel object and mice were allowed to explore both the familiar and novel object for a total of 30 min. Measurements of time spent with each object were scored during min 2 through 12 of the acquisition and trial phase. The Novel Object Recognition Index was calculated as the (Time spent with novel object/(Time spent with novel object + Time spent with familiar object)). Object interactions were defined as active interaction with the object where the mouse's nose was at least 1 cm pointed toward the object, time spent interacting/touching, and active sniffing of the object. Rearing on the objects was not scored. Exclusion criteria was set for <30 s interaction with both objects during the acquisition phase. One WT and two ArcKR mice did not meet the criteria and were excluded from the analysis.

Barnes maze test

WT (24) and ArcKR male mice (27) aged 21-25 days were tested. Out of these 5 WT and 6 ArcKR mice were trained for 1 day, 5 WT and 7 ArcKR mice were trained for 15 days and 19 WT and 14 ArcKR were trained for 20-21 days. Spatial learning was assessed using a modified circular Barnes maze that measured 1 m in diameter, was situated 1 m from the floor, and contained 20 5-cm holes that were evenly spaced (5 cm) around the perimeter. The maze was positioned centrally within the lab, with surrounding equipment and architectural features kept in fixed positions, to act as spatial cues for learning. The maze contained an 'exit' box positioned under one of the holes and a "fake" box (incorporated to mimic light reflection from the exit box but with no depth) under another. The exit hole was randomly assigned on the first day and maintained in this position for 15 days for the "exit" box prior to the 180° shift (see below) and for the remaining 5-6 days of training. Each day, mice were randomly placed in the center of the maze, released, and allowed to

explore the maze. The task was completed when the mouse entered the exit box. All runs were recorded using a camera system (Henelec Model 335 BWL SONY) attached to a computer for offline analysis (Any-MAZE v4.96, Stoelting). Total distance, speed, and accuracy of task performance were measured. On days 1-5, the exit box contained flavored treats as a reward for task completion. On days 6-21, treats were awarded in the home cage on completion to prevent cued orientation of the exit box location via olfactory stimulation. On days 16-21, the position of the exit box was rotated 180° to determine the spatial component of and coping ability for the task. Data analysis was carried out in a blind fashion, independently from the experimenter. Error number was measured by calculating the number of incorrect holes visited before locating the correct "exit" hole. Mapping the progression of the animals around the maze allowed determination of the search strategy. These were: random: no consistent pattern, >2 crossings of the open field; serial: a hole-by-hole progression with ≥ 3 consecutive holes visited; and spatial: moving directly to the exit hole ± 2 holes and no deviation outside of the quadrant.

Electrophysiology

AMPA receptor-mediated-miniature excitatory postsynaptic currents (mEPSCs) recorded in cultured neurons

Hippocampal neuronal cultures were prepared from postnatal day 0 pups from C57BL/6 wild-type mice as previously described (Corrêa et al., 2009). Briefly, hippocampi were dissected from the brain, dissociated with trypsin, and approximately 10^5 cells were plated onto 22-mm glass coverslips coated with poly-L-lysine in Neurobasal medium containing 1% L-glutamine, 1% penicillin-streptomycin, 2% B27 supplement (Invitrogen) and 5% horse serum. 24 hr after plating the media was completely changed and cells were grown in serum-free media. Cultures were maintained at 37°C and 5% CO_2 in a humidified incubator and transfections of either scrambled or Triad3-shRNA (Mabb et al., 2014a) were performed using Lipofectamine 2000 (Invitrogen). Cells expressing scrambled or Triad3-shRNA constructs were recorded at least 3-5 days after transfections. Coverslips were transferred to the recording chamber and perfused at a constant flow rate of (2 mL/min) with recording solution composed of (mM): 127 NaCl, 1.9 KCl, 1 $MgCl_2$, 2 $CaCl_2$, 1.3 K H_2PO_4, 26 $NaHCO_3$, 10 D-glucose, pH 7.4 (when bubbled with 95% O_2 and 5% CO_2, 300 mOsm) at 28-30°C. Tetrodotoxin (Tocris, 1 μM), picrotoxin (50 μM, Tocris), and L-689,560 (5 μM, Tocris) were present in the recording solutions to isolate mEPSCs. To induce mGluR-dependent synaptic depression, (RS)-3,5-dihydroxyphenylglycine, (DHPG, 100 μM, Tocris) was bath applied for 10 min. Neurons were visualized using IR-DIC optics with an Olympus BX51W1 microscope and Hitachi CCD camera (Scientifica) at a total magnification of 400X. Whole-cell patch clamp recordings were made from transfected (identified by fluorescence at 488 nm) pyramidal neurons using patch pipettes (5-8 MΩ) made from thick walled borosilicate glass (Harvard Apparatus) filled with (mM): 135 potassium gluconate, 7 NaCl, 10 HEPES, 0.5 EGTA, 10 phosphocreatine, 2 MgATP, 0.3 NaGTP, pH 7.2, 290 mOsm. Recordings of mEPSCs were obtained at a holding potential of −75 mV using an Axon Multiclamp 700B amplifier (Molecular Devices), filtered at 3 kHz and digitized at 20 kHz (Digidata 1440A, Molecular Devices). Data acquisition was performed using pClamp 10 (Molecular Devices). Analysis of mEPSCs was performed using MiniAnalysis software (SynaptoSoft). Events were manually analyzed and were accepted if they had an amplitude >5 pA (events below this amplitude were difficult to distinguish from baseline noise) and a faster rise than decay. Statistical significance was measured using a one-way ANOVA with 0.05% taken as significant.

Hippocampal slice preparation

Hippocampal slices (400 μm) were obtained from 21 to 35 day-old WT and ArcKR littermates. For trained mice, slices were obtained up to 1 hr after the last training session. Animals were sacrificed by cervical dislocation and decapitated in accordance with the UK Animals (Scientific Procedures) Act (1986). The brain was rapidly removed and placed in ice-cold high Mg^{2+}, low Ca^{2+} artificial CSF (aCSF), consisting of the following (in mM): 127 NaCl, 1.9 KCl, 8 $MgCl_2$, 0.5 $CaCl_2$, 1.2 KH_2PO_4, 26 $NaHCO_3$, 10 D-glucose (pH 7.4 when bubbled with 95% O_2 and 5% CO_2, 300 mOSM). Parasagittal brain slices were then prepared using a Microm HM 650V microslicer in ice-cold aCSF (2-4°C). Slices were trimmed and the CA3 region was removed. Slices were allowed to recover at 34°C for 3-6 hr in aCSF (1 mM $MgCl_2$, 2 mM $CaCl_2$) before use.

Extracellular recording from hippocampal slices

Field excitatory postsynaptic potentials (fEPSPs) were recorded from interleaved slices from WT and ArcKR littermates. An individual slice was transferred to the recording chamber, submerged in aCSF (composition as above), maintained at 32°C, and perfused at a rate of 6 mL/min. The slice was placed on a grid allowing perfusion above and below the tissue and all tubing was gas tight (to prevent loss of oxygen). To record fEPSPs, an aCSF filled microelectrode was placed on the surface of stratum radiatum in the CA1 region. A bipolar concentric stimulating electrode (FHC) controlled by an isolated pulse stimulator, model 2100 (AM Systems, WA) was used to evoke fEPSPs at the Schaffer collateral–commissural pathway. All recordings were made in the presence of 50 μM picrotoxin to block $GABA_A$ receptors (Tocris) and the NMDA receptor antagonist L-689,560 (trans-2-carboxy-5,7-dichloro-4-phenylaminocarbonylamino-1,2,3,4-tetrahydroquinoline; 5 μM; Tocris). Field EPSPs were evoked at 0.1 Hz (200 μs stimulus), with a 20-min baseline recorded at a stimulus intensity that gave 40% of the maximal response. To induce mGluR-LTD, 50 or 100 μM of (RS)-3,5-DHPG (3,5-dihydroxyphenylglycine, Tocris) was applied for 10 min and then washed off for at least one hour as previously described (Eales et al., 2014). Recordings of fEPSPs were made using a differential model 3000 amplifier (AM systems, WA USA) with signals filtered at 3 kHz and digitized online (10 kHz) with a Micro CED (Mark 2) interface controlled by Spike software (Vs 7.08), Cambridge Electronic

Design, Cambridge UK). Field EPSPs were analyzed using Spike software and graphs prepared using Origin (Microcal), with the slope of fEPSPs measured for a 1 ms linear region following the fiber volley.

Statistical Analysis

Statistical analyses applied were the post hoc Student's t test or repeated-measures ANOVA with pairwise multiple comparisons. Behavioral data were analyzed using two-way or repeated-measures Analysis of Variance (ANOVA). Fisher's protected least-significant difference (PLSD) tests were used for comparing group means only when a significant F value was determined. For all comparisons, significance was set at $p \leq 0.05$. Data presented in figures and tables are means (±SEM).

Neuron, Volume *98*

Supplemental Information

The Temporal Dynamics

of Arc Expression

Regulate Cognitive Flexibility

Mark J. Wall, Dawn R. Collins, Samantha L. Chery, Zachary D. Allen, Elissa D. Pastuzyn, Arlene J. George, Viktoriya D. Nikolova, Sheryl S. Moy, Benjamin D. Philpot, Jason D. Shepherd, Jürgen Müller, Michael D. Ehlers, Angela M. Mabb, and Sonia A.L. Corrêa

Figure S1

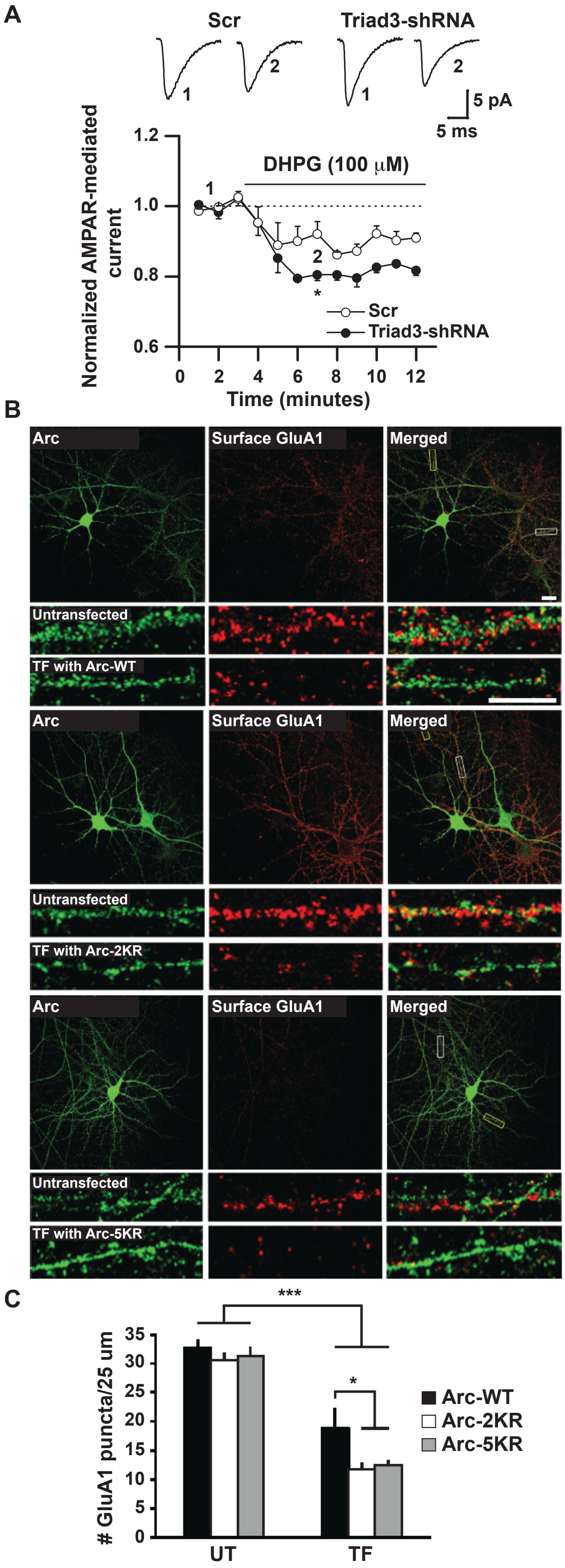
A
Scr
Triad3-shRNA
1
2
1
2
5 pA
5 ms
Normalized AMPAR-mediated current
1.2
1.0
0.8
0.6
DHPG (100 μM)
1
2
*
Scr
Triad3-shRNA
0
2
4
6
8
10
12
Time (minutes)
B
Arc
Surface GluA1
Merged
Untransfected
TF with Arc-WT
Arc
Surface GluA1
Merged
Untransfected
TF with Arc-2KR
Arc
Surface GluA1
Merged
Untransfected
TF with Arc-5KR
C
***
*
GluA1 puncta/25 um
35
30
25
20
15
10
5
0
UT
TF
Arc-WT
Arc-2KR
Arc-5KR

Figure S1. ArcKR expression enhances GluA1 internalization, Related to Figure 1

(A) Graph plotting normalized mean AMPA receptor-dependent mEPSC amplitudes against time for hippocampal cultured neurons expressing either *Triad3/Rnf216*-shRNA (n = 5) or scrambled RNA (n = 4) from at least 2 different culture preparations. Treatment with DHPG (100 μM) significantly enhances DHPG-LTD in *Triad3/Rnf216*-shRNA compared to neurons expressing scrambled RNA (measured at 6-7 min in DHPG compared to baseline, p = 0.026). *Top*, representative averaged mEPSC waveforms recorded at baseline and during DHPG exposure at times indicated in the plot (1, 1-2 min and 2, 6-7 min). Averages were constructed from at least 50 mEPSCs which were aligned on 50% of the rising phase. (B) Cultured cortical neurons (DIV 15) were transfected with Arc-WT (upper panel), Arc-2KR (middle), or Arc-5KR (bottom) constructs. Neurons were stained for surface GluA1 and Arc 16 h after transfection. As expected, overexpression of Arc-WT reduced surface GluA1 puncta compared to untransfected neurons. Zoomed panels, dendrites from untransfected (white box) and transfected (yellow box) neurons. Scale bars = 10 μm. (C) Overexpression of the Arc-KR constructs resulted in significant reduction in surface GluA1 puncta compared to Arc-WT overexpression. *n* = 2 dendrites/neuron, 15 dendrites/group. *p < 0.05; ***p < 0.001. Values represent mean ± S.E.M.

Figure S2

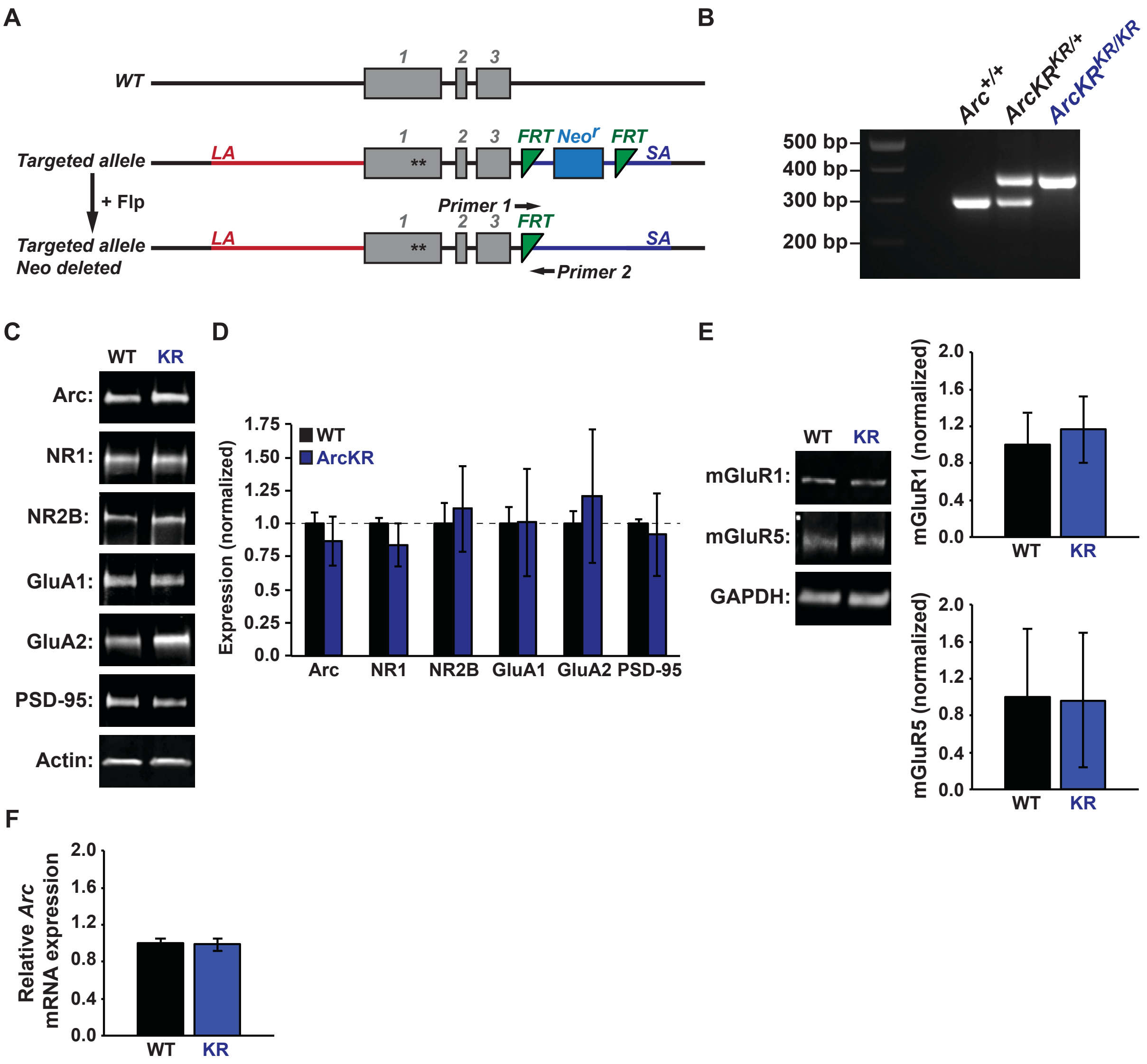

A
WT
1
2
3
Targeted allele
LA
**
FRT
Neo^r
FRT
SA
+ Flp
Primer 1
Targeted allele
Neo deleted
Primer 2
B
Arc+/+
ArcKR KR/+
ArcKR KR/KR
500 bp
400 bp
300 bp
200 bp
C
WT
KR
Arc:
NR1:
NR2B:
GluA1:
GluA2:
PSD-95:
Actin:
D
WT
ArcKR
Expression (normalized)
1.75
1.50
1.25
1.0
0.75
0.5
0.25
0.0
Arc
NR1
NR2B
GluA1
GluA2
PSD-95
E
mGluR1:
mGluR5:
GAPDH:
mGluR1 (normalized)
mGluR5 (normalized)
2.0
1.6
1.2
0.8
0.4
0.0
F
Relative Arc mRNA expression

Figure S2. Generation and characterization of the ArcKR mouse, Related to Figure 1

(A) Schematic of targeting strategy used to generate Arc knock-in mice. Primer positions represent sites used to distinguish wildtype ($Arc^{+/+}$; WT), heterozygous ($Arc^{KR/+}$; HET), and homozygous ($Arc^{KR/KR}$; ArcKR) mice. (B) Genotyping results from WT, HET, and ArcKR mice. Wildtype band size is 291 base pairs and the knock-in band size is 355 base pairs. (C) ArcKR neurons do not exhibit synaptic abnormalities. Western blot of synaptosome fractions isolated from WT or ArcKR mice. Actin was used as a loading control. (D) Quantification of synaptic protein expression in WT and ArcKR mice. All proteins are normalized to Actin. (E) Western blot of hippocampi isolated from postnatal day 60 (P60) WT or ArcKR mice. GAPDH was used as a loading control. n = 3 independent experiments. (F) Quantitative pPCR demonstrating no change in *Arc* mRNA expression between WT and ArcKR mice at P60. Hippocampi from WT and ArcKR mice were collected and the total RNA was isolated. *Arc* mRNA was determined by quantitative PCR. Data were normalized to the geometric mean of *GAPDH* reference gene and *Arc*. Each data point represents duplicate measurements from an individual mouse from 12 WT and 9 ArcKR mice. Values represent mean ± S.E.M.

Figure S3

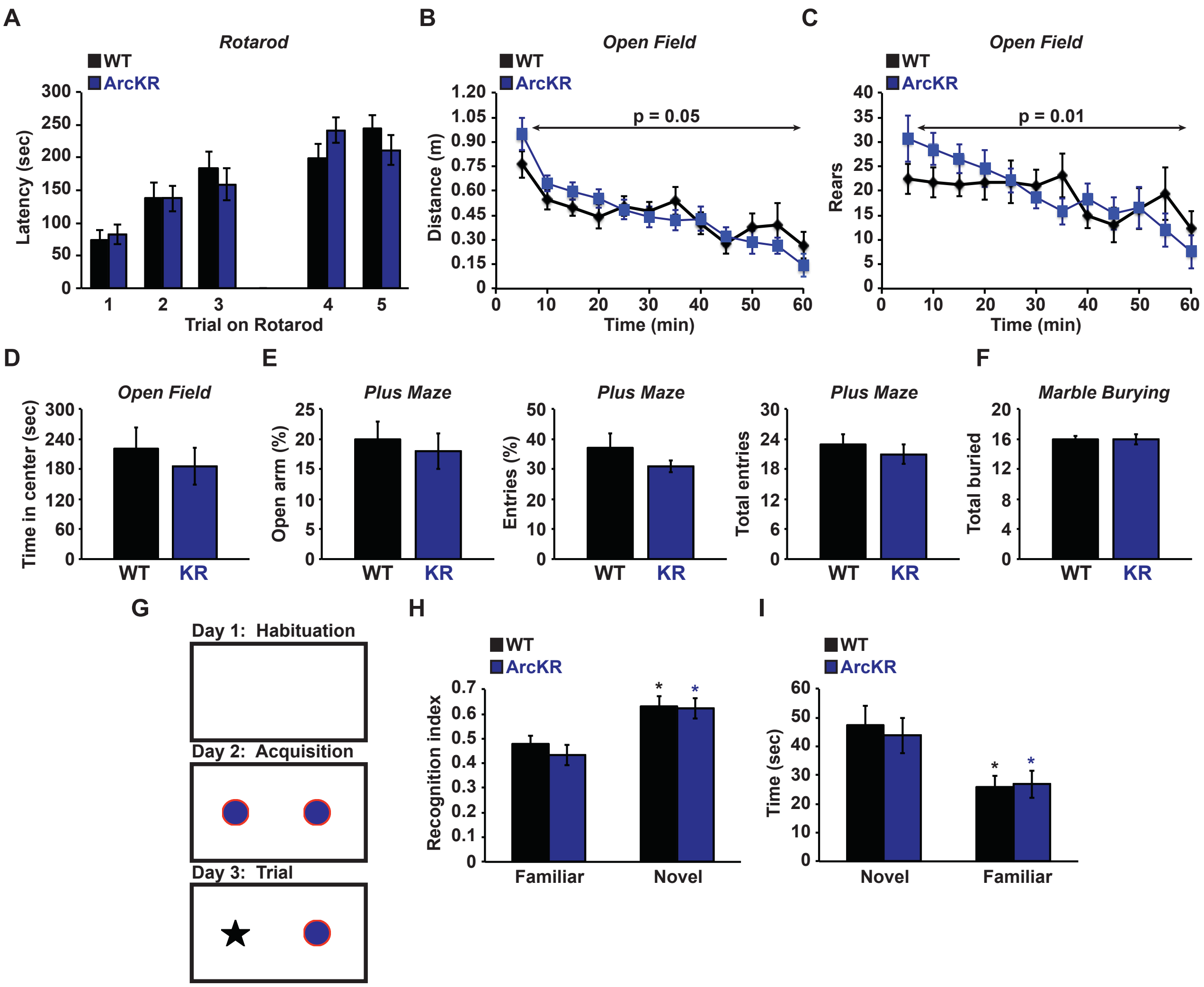
A
Rotarod
WT
ArcKR
Latency (sec)
Trial on Rotarod
B
Open Field
WT
ArcKR
Distance (m)
p = 0.05
Time (min)
C
Open Field
WT
ArcKR
Rears
p = 0.01
Time (min)
D
Open Field
Time in center (sec)
WT
KR
E
Plus Maze
Open arm (%)
Plus Maze
Entries (%)
Plus Maze
Total entries
F
Marble Burying
Total buried
G
Day 1: Habituation
Day 2: Acquisition
Day 3: Trial
H
WT
ArcKR
Recognition index
Familiar
Novel
I
WT
ArcKR
Time (sec)
Novel
Familiar

Figure S3. Behavioral characterization of ArcKR mice, Related to Figure 3

(A) Motor coordination and learning are unaltered in ArcKR mice. Graph plotting the time until mice fall off an accelerating rotarod. Mice were given three trials on day 1 (1, 2, and 3). Mice were then given 2 additional trials 48 hours later (4 and 5). (B) Initial locomotor activity in a novel open field is enhanced in ArcKR mice. Distance traveled over a time course of 60 min after placement in a novel environment. (C) Initial rearing activity in a novel open field is enhanced in ArcKR mice. Number of rears were taken within 60 min after placement in a novel environment. (D) ArcKR mice spend similar time in the center as WT mice in a novel open field. Time spent in the center of a novel open field within 60 min after placement in a novel environment. (E) ArcKR mice exhibit normal anxiety-like behavior in an elevated plus maze. Percent time spent in open arm (left), percent entries (middle), and total number of entries (right) in WT and ArcKR mice. (F) ArcKR mice exhibit normal marble burying behavior. (G) ArcKR mice have normal long-term recognition memory. Experimental setup for the Novel Object Recognition Test. (H) Novel Object Recognition Index values of WT and ArcKR mice. WT = 11 animals, ArcKR = 10 animals. (I) Total time mice spent with the Novel versus Familiar Object in the Trial phase of the NORT. Values represent mean ± S.E.M, $p \leq 0.05$. WT=12 animals and ArcKR-12 animals

Figure S4

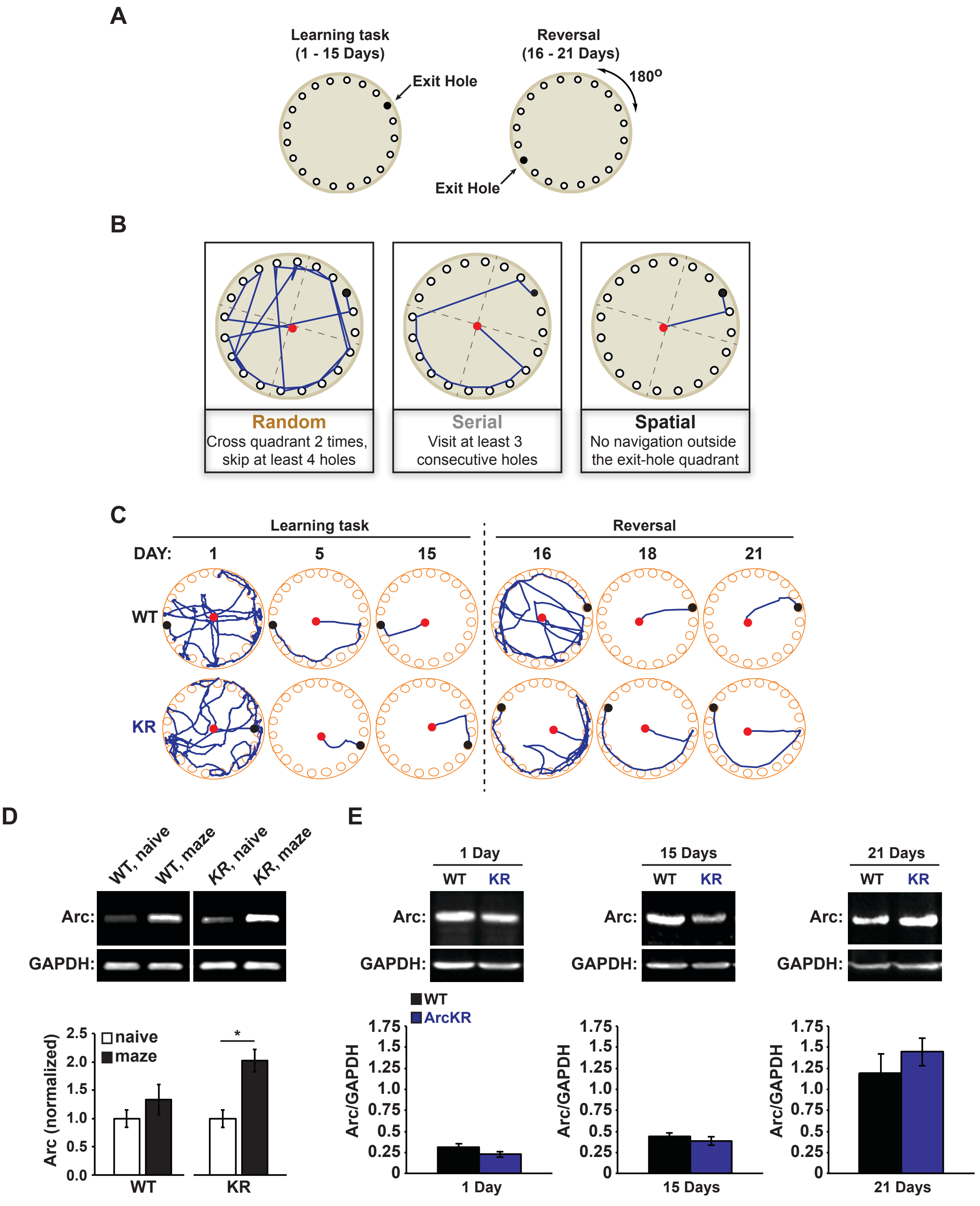
A
Learning task
(1 - 15 Days)
Exit Hole
Reversal
(16 - 21 Days)
180°
Exit Hole
B
Random
Cross quadrant 2 times, skip at least 4 holes
Serial
Visit at least 3 consecutive holes
Spatial
No navigation outside the exit-hole quadrant
C
Learning task
Reversal
DAY: 1 5 15 16 18 21
WT
KR
D
WT, naive
WT, maze
KR, naive
KR, maze
Arc:
GAPDH:
Arc (normalized)
naive
maze
*
WT
KR
E
1 Day
15 Days
21 Days
WT KR
Arc:
GAPDH:
WT
ArcKR
Arc/GAPDH
1 Day
15 Days
21 Days

Figure S4. Arc knock-in mice exhibit deficits in cognitive flexibility, Related to Figure 3
(A) Schematic drawing showing the Barnes Maze experimental setup. Mice were tested a single time daily for 15 days. On day 16, the maze was rotated 180 degrees, mice were tested a single time daily, as before. (B) The scoring parameters used to assess learning strategies for the Barnes Maze paradigm. (C) Representative activity traces in WT and ArcKR mice during days 1, 5, 15, 16, 18, and 21. (D) *Top*, blot showing Arc protein levels obtained from hippocampal lysate of Barnes maze trained WT and ArcKR mice. Actin was used as a loading control. *Bottom*, Graph represents Arc levels normalized to Actin from D. N = 3, $p \leq 0.05$. (E) Blots showing Arc protein obtained from hippocampal lysates of Barnes maze WT and ArcKR mice trained at 1, 15, and 21 days (WT 1day: n=5; WT 15 days: n=5; WT 21 days: n = 4; ArcKR 1 day: n=6; ArcKR 15 days: n=7; ArcKR 21 days: n = 5). GAPDH was used as a loading control. Graphs represent Arc levels normalized to GAPDH. Values represent mean ± S.E.M.
